# Emotion regulation in mother-child dyads is associated with interbrain synchrony during imagined shared emotional experiences

**DOI:** 10.3389/fpsyg.2026.1813005

**Published:** 2026-07-01

**Authors:** Inês Rodrigues, Diana Costa, João Pereira, Rita Correia, Pascal Vrtička, Teresa Sousa, Miguel Castelo-Branco

**Affiliations:** 1 Coimbra Institute for Biomedical Imaging and Translational Research (CIBIT), University of Coimbra, Coimbra, Portugal; 2 Institute for Nuclear Sciences Applied to Health (ICNAS), University of Coimbra, Coimbra, Portugal; 3 Faculty of Medicine, Institute for Physiology, University of Coimbra, Coimbra, Portugal; 4 Intelligent Systems Associate Laboratory (LASI), Guimarães, Portugal; 5 Department of Psychology, Centre for Brain Science, University of Essex, Colchester, United Kingdom

**Keywords:** emotion regulation, fNIRS, hyperscanning, mother-child dyads, relational neuroscience

## Abstract

Parents play an important role in the development of children's emotion regulation (ER) skills—not only through their responses to children's emotions themselves, but also as role models by demonstrating their own ER strategies. Recent studies suggest that interbrain synchrony (IBS) can provide deeper insights into such parent-child dynamics at the neural level. IBS has been suggested as a neural marker of interpersonal processes such as bond formation and information exchange, and is known to vary by emotional valence and social context. This work aimed to investigate IBS during shared mother-child emotional experiences across different social contexts to study the neural mechanisms underlying mothers' and children's ER skills. An emotional imagery task, in which 36 mother-child dyads (child age 10–14 years) imagined experiencing emotional situations both *with* and *without each other*, was applied. Brain activity was simultaneously recorded using functional near-infrared spectroscopy (fNIRS) over the right frontopolar cortex, dorsolateral prefrontal cortex (rdlPFC) and temporoparietal junction (rTPJ). Results revealed that for all the regions analysed IBS only varied across valences in the *with each other* social condition, with higher IBS observed in negative and neutral scenarios compared to positive ones. Furthermore, IBS in the *with each other* social condition was positively associated with children's use of cognitive reappraisal (i.e., psychological reframing situations) across valences. For the negative valence, IBS also showed a positive association with mothers' ER difficulties. These findings suggest that dyadic IBS might reflect personal traits related to ER and provide a foundation for future investigations into the neural mechanisms underlying ER development.

## 1 Introduction

Emotions subserve inner responses to various sources of information that can help navigate our environment and social interactions. However, when not adequate to the moment, emotions can become maladaptive, highlighting the importance of emotion regulation (ER) (Gross, 2014). ER refers to the process through which individuals can adapt their emotional reactions in intensity and/or duration (Cole et al., 2004) as well as their ability to recognise and respond to others' emotions (Ratliff et al., 2022).

Various ER strategies can be employed, including, amongst others cognitive reappraisal and expressive suppression. The former refers to cognitively reframing situations to alter their emotional impact and is generally associated with more positive and less negative affect. Conversely, the latter involves hiding or inhibiting the outward expression of emotions as a response-focused strategy, which can result in greater psychological costs like less positive affect and diminished impact on the control of negative experiences (Gullone and Taffe, 2012). Poorer ER skills are usually linked to a range of mental health issues, including depression and anxiety in both adolescents and adults (Kaufman et al., 2016; Silk et al., 2003), and externalising behaviors (e.g., lying or stealing) in adolescents (Silk et al., 2003).

ER skills begin to develop during childhood and are influenced by early relational experiences (Morris et al., 2017; Ratliff et al., 2022). Parents' behavior toward their children in emotional situations, as well as during the parents' expression of their own emotional experiences, serves as a model through which children learn to understand and deal with emotions (Morris et al., 2017). Although children's social circle expands during pre-adolescence (ages 10 to 14), parents remain their primary caregivers, with children showing a continuing tendency to seek comfort from them during distress. Similarly, children still count on their parents for support when exploring novel physical, social, and emotional environments and to share their experiences thereof (Koehn and Kerns, 2016; Ratliff et al., 2022).

Given the importance of the parent-child relationship in development, including ER skills, it is important to understand not only the psychological and behavioral aspects of these dyads dynamics, but also the neurobiological processes that support them. Recently, this has been studied based on interbrain synchrony (IBS) (e.g., Reindl et al., 2018). IBS refers to the temporal alignment of brain activity that occurs during or shortly after social encounters and experiences. It is one of the components of bio-behavioral synchrony (BBS)and is hypothesised to be stronger between individuals who share closer/more intimate bonds (De Felice et al., 2024; Feldman, 2017; Gvirts and Perlmutter, 2020).

In parent-child dynamics, IBS modulation has been observed across different contexts. For example, in studies where dyads are physically present in the same room but only engage in tasks involving minimal social interaction (De Felice et al., 2024), IBS was found to be increased during father-child watching of videos containing information with higher levels of arousal (Azhari et al., 2021). In another study using a similar co-watching setup, the results suggested that mother-child IBS was negatively associated with mothers' attachment anxiety (Azhari et al., 2023). Besides co-presence studies, IBS has also been found to vary in parent-child dyads performing interactive tasks. For instance, in cooperation tasks, researchers observed that mother-child and father-child dyads exhibited greater IBS when collaboratively solving tangram puzzles compared to completing puzzles individually (Nguyen et al., 2024). Similarly, parent-child dyads exhibited greater IBS in a computer-based button press task when cooperating than when competing with each other (Reindl et al., 2018) or performing the task individually (Miller et al., 2019). In another study, when parent–child dyads were prevented from accessing desirable toys while completing a challenging tangram puzzle (frustration condition), they exhibited higher IBS than during a baseline art project or a post-frustration free-play condition in which they were allowed to engage with the toys (Thompson et al., 2024). Taken together, these findings support the hypothesis that parent-child interaction dynamics, such as for bond-formation from early developmental stages (Feldman, 2017), might be studied at the neural level through IBS.

Importantly, IBS has also been observed to vary depending on the emotional content of the performed experimental tasks by the dyads. In a mother-child (10 to 42 months old) naturalistic play task, researchers observed greater IBS during moments when mother and child shared high positive affect, compared with moments of shared low positive affect, particularly in brain regions related to emotion and mentalising (Morgan et al., 2023). In adult dyads, during a task wher participants shared negative memories with a stranger, increased IBS was observed as compared to a control condition (Wang et al., 2022). While this study considered IBS when only one participant shared their emotional experiences with another, variations in IBS have also been observed when both participants were equally engaged with the emotional task content. In another study where different types of adult dyads were asked to discuss both supportive and conflict topics, IBS was greater for conflict topics in romantic partner dyads but greater for supportive topics in friend dyads (Long et al., 2022). A previous study IBS variation for films of different emotional content (Nummenmaa et al., 2012). The findings showed that IBS was higher when participants watched films with negative compared to positive content. In a task where participants read/listened to emotional stories, the direction of the association between IBS and stimulus valence has been found to vary depending on the brain region evaluated. Particularly, positive associations were observed in the bilateral temporal pole and left posterior superior temporal sulcus (pSTS), regions involved in emotional processing and assessment of intention, respectively. A negative association was found in the bilateral superior frontal gyrus, a region involved in mentalizing, and right pSTS (Smirnov et al., 2019).

IBS has been shown to vary not only with emotional valence but also with social context. In a study, in which IBS was evaluated in mother-father dyads during the listening to emotional audio stimuli (positive, negative, and neutral) either together in the same room or in separate rooms, IBS was found to be higher during positive and neutral stimuli when dyads were in the same room (Azhari et al., 2020). These findings highlight that similar emotional experiences might be processed differently depending on the social context. Nevertheless, further research is still needed to better understand such neural processes when participants are actively experiencing emotional events.

Aside from the emotional content of the task/stimuli, IBS can also translate participants' emotional state. In a previous study in which mother-child dyads co-watched animation videos, IBS derived from the middle PFC was negatively associated with mothers' self-reported stress levels. These findings were hypothesised to be related to worse dyad' engagement quality or reduced mentalising capacities (Azhari et al., 2019). In another study, during parent-child joint free play, researchers observed that the direction of the association between IBS and parental stress appeared to vary depending on the brain region (Azhari et al., 2022). They found a positive association in the anterior prefrontal cortex's (PFC) frontal areas, areas related to attention and planning, but a negative association with the PFC's posterior areas, related with ER and selective attention. These findings might point for neural evidence of lower cognitive effort for engagement in shared play in dyads with less stressed parents (Azhari et al., 2022). Finally, previous research has also investigated the direct association between IBS and ER skills. Using a computer-based button press task, Reindl and colleagues (2018) observed that IBS in parent-child dyads during cooperation (compared to competition) was positively associated with both parents' and children's ER skills. These findings suggest that these factors may similarly shape the neural mechanisms underlying the perception of shared experiences by each dyad member and affect the quality and cognitive effort needed for dyadic engagement. However, how ER skills are related to IBS during tasks in which the participants undergo more complex and direct emotional experiences remains to be investigated.

In line with that, our study investigated IBS between mothers and their pre-adolescent children during imagined emotional experiences varying in valence (positive, negative and neutral) and social context (situations imagined to be experienced either together or apart). Emotional imagery refers to the simulation of emotionally salient experiences via internally generated mental representations (Cocquyt and Palombo, 2023). Evidence from previous research indicates that emotional mental imagery can produce neural effects comparable to those of real-life emotional experiences (Holmes et al., 2008; Rainville et al., 2006; Travassos et al., 2020, for a review, see Ji et al., 2016), making it an effective strategy for studying emotional-related neural mechanisms during different experience valences (positive, negative and neutral). We focused on mother-child dyads imagining the experience of emotional situations, given the important role parents play in the development of children's ER skills (Morris et al., 2017; Ratliff et al., 2022). Our aim was to test the hypothesis that mother-child IBS varies depending on valence and social context of each experienced situation, acting as a potential marker of the neurobehavioral mechanism contributing for the dyadic interaction, in particular the ones linked to ER.

Previous research has suggested that emotional mental imagery elicits activation in brain regions involved in attentional and emotional processing, including limbic and prefrontal systems (Costa et al., 2010; Ji et al., 2016; Kosslyn et al., 2001). By asking to imagine shared experiences, we follow the hypothesis that the social context (together vs. apart) (e.g., Azhari et al., 2020; Miura and Noguchi, 2022), can also be reflected on these neural systems. We focused on the frontopolar cortex, the dorsolateral prefrontal cortex (dlPFC), and the temporoparietal junction (TPJ) due to their known roles in emotion and mentalizing. The frontopolar and dlPFC regions have been associated with emotion processing and regulation (Feldman, 2015; Lindquist et al., 2016; Raposo et al., 2011), whereas the TPJ has been implicated in theory of the mind and mentalizing processes (Feldman, 2015; Gvirts and Perlmutter, 2020; Long et al., 2020; Stietz et al., 2019). Furthermore, these regions have been also associated with significant IBS modulation during the discussion of emotional topics (Wang et al., 2022) and with ER skills (Reindl et al., 2018).

Given the association between IBS and personal traits (De Felice et al., 2024), particularly within parent-child dyads in relation to individual differences in ER skills (Reindl et al., 2018), we also aimed to investigate whether IBS during shared imagined experiences was related to different aspects of emotion regulation. Particularly, we investigated whether IBS during situations imagined *with each other* varied as a function of children's use of different ER strategies, testing the hypothesis that IBS modulation during emotional shared experiences is linked to children's use of ER strategies. Additionally, we investigated the association between IBS during negative situations imagined *with each other* and mothers' ER difficulties, which are conceptualised as reflecting emotional regulation challenges (Kaufman et al., 2016; Moreira et al., 2022) to test the hypothesis that IBS modulation by emotional experiences is associated with mothers' ER difficulties.

## 2 Methods

### 2.1 Participants

41 dyads of mothers with their biological children were recruited. Mothers' age varied between 35, and 56 years (M_mothers' age_ = 46.444, S.D._mothers' age_ = 4.164), and children's age between 10 and 14 years (n_males_ = 17, M_children's age_ = 12.556, S.D._children's age_ = 1.027). Due to the fNIRS data quality criteria adopted (detailed below), we excluded data from five dyads. 36 dyads (35 mothers, as one mother participated twice, once with each twin child) were therefore included in the final data analysis. All participants were Portuguese speakers, healthy and with normative development. Among children, 25% had no siblings, and 94% of all participants were right-handed. Additional inclusion criteria included absence of any history of brain trauma, psychiatric disorders or substance abuse, as well as no current diagnosis of psychological disorders. The current study received approval from the Ethics Committee of the Faculty of Medicine of the University of Coimbra (reference CE-099/2020). In addition to providing verbal consent, all participants received and signed a written informed consent form. Mothers signed their own as well as their children's consent forms.

### 2.2 Procedure and set-up

**Figure 1:**
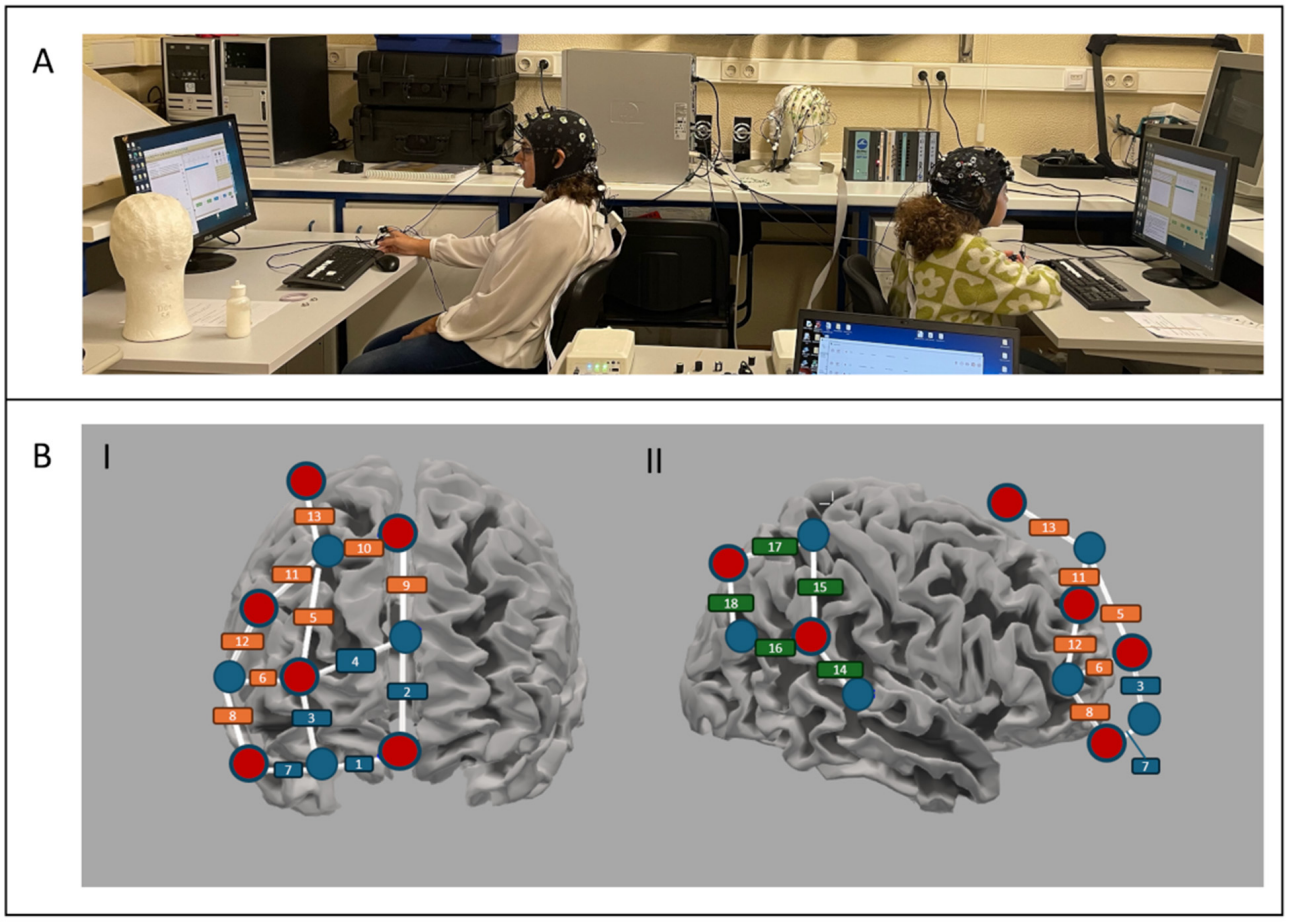
Data acquisition set-up and fNIRS template configuration. **(a)** Task set-up. Participants sat at individual desks facing individual monitor screens, with their backs to each other. **(b)** fNIRS illustration of optode and channel placement. The anterior view is presented on the left **(I)** and the right lateral view on the right (II). Red dots represent emitters, blue circles around red dots represent short-channels, and blue dots represent detectors. Channels formed by emitter-detector pairs are represented by numbers inside squares, where the orange squares represent channels in the dlPFC, blue squares represent channels in the frontopolar cortex, and green squares represent channels in the TPJ (Rodrigues et al., 2026), [Creative Commons Attribution 4.0 International License].

Participants were invited to the laboratory for a session lasting approximately two hours. During the visit, each participant filled out questionnaires (demographic data and psychological scales, see below), was fitted with an fNIRS cap and electrodermal activity (EDA) electrodes (see Supplementary methods and Supplementary Results), and completed two emotional imagery tasks (differing on who was the direct target of the emotion) while wearing the fNIRS and EDA sensors. The present manuscript only reports data from one task, results from the other task are reported elsewhere (Rodrigues et al., 2026). Before completing the emotional imagery task, participants were provided a detailed explanation using real examples drawn from the task itself. While filling out the questionnaires and completing the imagery task, participants sat back-to-back, with 170 cm separating their desks, preventing them from seeing each other. During the task, the same stimuli were presented simultaneously to each participant on individual computer screens (viewing distance = 55 cm, visual angle = 51.45°). Participants used keyboards to rate the valence of each imagery moment (Figure 1A).

### 2.3 Task

**Figure 2:**
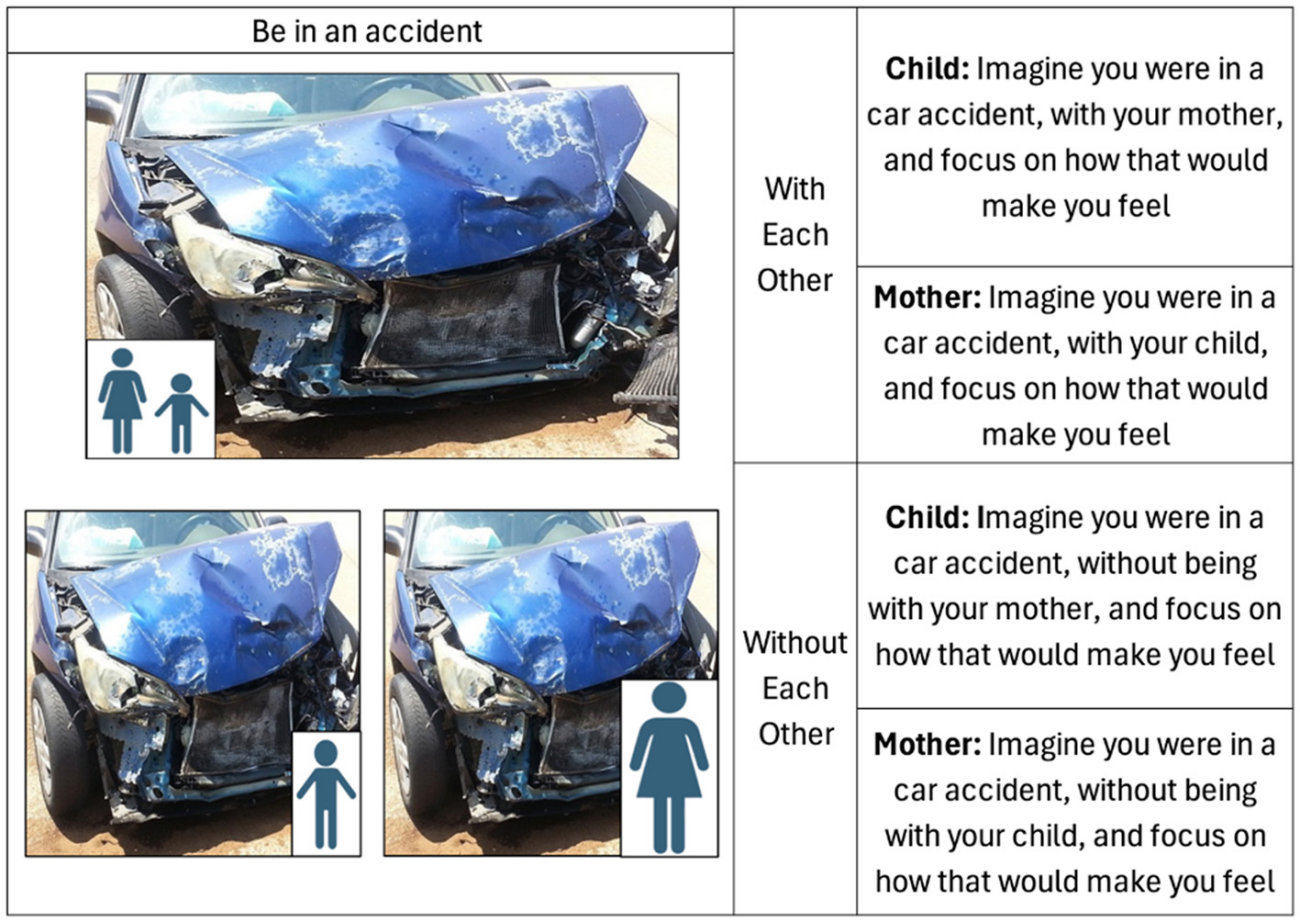
Schematic representation of the emotional imagery task. Example of a negative valence scenario (picture car accident 2 from the OASIS database (Kurdi et al., 2017) labelled as “Be in an accident.” The top row represents the with each other social condition, and the bottom row the without each other social condition. On the right side is an example of the task instructions adapted to this example (the task general instructions were provided before the task).

Participants performed an emotional imagery task in which they were asked to imagine different situations with both of them being the direct target. The task included situations from three valence types (positive, negative, and neutral) which participants were instructed to imagine under two distinct social conditions: *with each other* and *without each other*. In the *with each other* social condition, both participants were asked to imagine experiencing the presented situations together (*i.e*., children with their mothers and vice versa) and to focus on how this shared experience would make them feel. In the *without each other* condition, both participants were asked to imagine experiencing the presented situations by themselves (*i.e*., without the other participant being present) and to focus on how the situation would make them feel. For example, one of the negative scenarios instructed participants to imagine being in a car accident and was presented with the label “Be in an accident,” accompanied by a picture. In the *with each other* condition, both participants were asked, for example, to imagine being in a car accident together (i.e., children with their mothers and vice versa) and to focus on how experiencing that situation together would make them feel. Conversely, in the *without each other* condition, both participants were asked to imagine being in a car accident by themselves and to focus on how this would make them feel (Figure 2). All images used for this task can be found in the Supplementary Materials, (Supplementary Figure 1).

**Figure 3:**
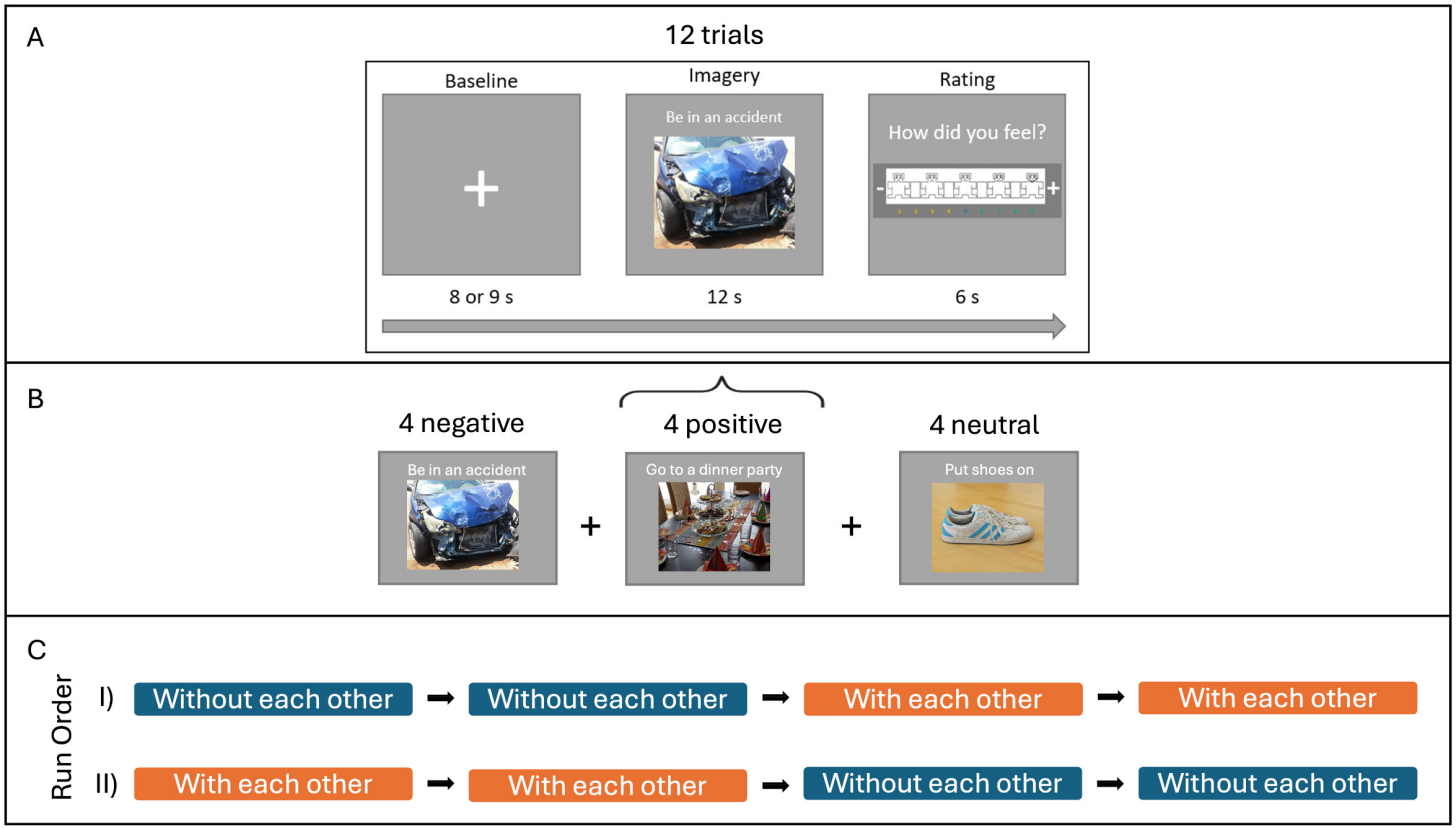
Schematic representation of the task. Each mother-child dyad completed four runs, two in each social condition. Stimuli order presentation **(c)** was counterbalanced, changing between order I) and II). Each run comprised twelve trials **(a)**, in a total of four imagery situations of each valence **(b)**. Pictures for the stimuli examples are derived from the OASIS (Kurdi et al., 2017) and things (Hebart et al., 2019) databases, and ratings were conducted using the SAM scale (Bradley and Lang, 1994; Lang, 1980).

The task consisted of multiple trials containing an imagery period of 12 s preceded by a fixation cross (for 8 or 9 s) and followed by a rating period (6 s). Each dyad completed 4 runs (2 from each social condition—alone or together), with each run including 12 imagery trials (four per valence—negative, neutral, or positive) presented in semi-randomised order (Figure 3). In total, each dyad performed 8 trials per valence type in each social condition, with the same set of scenarios used across both conditions. The same scenario was presented simultaneously to the dyad, and the order of the social conditions was counterbalanced across dyads (order I or II, Figure 3C). During each imagery period, a centred image (visual angle = 33.77° × 13.89°) accompanied by a descriptive label (visual angle = 4.19°) was used to direct participants on what they should imagine. Images were collected from the OASIS (Kurdi et al., 2017) and THINGS (Hebart et al., 2019) databases, both open sources, and consisted of colour photographs (all images scaled to 500 x 400 pixels, screen resolution = 1920 x 1080). Each imagery period was preceded by a baseline, comprising a fixation cross on a grey background (visual angle = 3.07°), and was followed up by a rating period (see below).

### 2.4 Behavioral measures

Subjective valence ratings were collected after each imagery period using the pictorial 9-point Self-Assessed Manikin (SAM) scale (Bradley and Lang, 1994; Lang, 1980) and were used as behavioral measures. For this study, each point in the SAM scale was numbered from 1 to 9, with 1 being the most negative feeling and 9 the most positive feeling, with 5 corresponding to neutral (Figure 3A). Individual behavioral measures were obtained by calculating the average subjective rating for each valence in each social condition (i.e., 3 valences x 2 social conditions), resulting in 6 behavioral measures per individual. Additionally, we derived two dyadic behavioral measures: dyadic average valence ratings and dyadic difference valence ratings. The first consisted of the mean of the mother's and child's average ratings, and the second the absolute value of the difference between the child's and the mother's average subjective valence rating for each valence. This procedure once again resulted in 6 dyadic behavioral outputs (3 valences x 2 social conditions) for each measure.

### 2.5 Psychological measures

To test the association between IBS and ER related processes, we assess ER dimensions in both mothers and children. Particularly, we measured mothers' difficulties in ER and children's use of different ER skills.

#### 2.5.1 Mothers

The Difficulties in Emotion Regulation Scale—Short Form (DERS-SF) (Kaufman et al., 2016; Moreira et al., 2022), a self-report questionnaire, was used to measure mothers' difficulties in ER (e.g., “When I'm upset, it takes me a long time to feel better”). The total score of the questionnaire was derived for analyses by averaging the scores of all items, excluding the items of the Awareness subscale, as recommended in the Portuguese adaptation of the scale (Moreira et al., 2022). A higher score in this questionnaire represents more pronounced difficulties in emotion regulation of negative affect.

#### 2.5.2 Children

The Emotion Regulation Questionnaire for Children and Adolescents (ERQ-CA) (Carvalho, 2014; Gullone and Taffe, 2012), a self-report questionnaire, was used to evaluate children's use of ER strategies. The ERQ-CA consists of two subscales: Cognitive Reappraisal (CR) and Expressive Suppression (ES). CR refers to changing the impact of an emotional situation by reframing the situation (item example: “When I want to feel happier, I think about something different”), while ES refers to inhibiting the expression of emotional behaviors as a strategy to deal with emotional events (item example: “I keep my feelings to myself”). Each subscale score results from adding up all individual items (CR's scores range from 6 to 30, and ES's scores range from 4 to 20). A higher score represents a greater use of the referred ER strategy.

### 2.6 fNIRS

#### 2.6.1 Optode placement and data collection

Brain activity was obtained simultaneously from mothers and children using two fNIRS devices (NIRSport2 continuous wave fNIRS system, NIRx Medical Technologies, Berlin) in hyperscanning mode. The sampling rate was set to 10.17 Hz, and the data were collected using the Aurora fNIRS acquisition software (Brain Innovation, the Netherlands). The optodes placement and ROI allocation (see Supplementary Table 1) were determined using the fOLD toolbox (version 2.2; Zimeo Morais et al., 2018), with optodes placed over the right frontopolar cortex, dlPFC, and TPJ. The fNIRS configuration used had a total of 18 long-distance channels distributed over the 3 ROIs (5 frontopolar, 8 dlPFC and 5 TPJ), and 8 short-distance channels (one for each emitter) (Figure 1B).

#### 2.6.2 fNIRS pre-processing and data quality assessment

fNIRS data from each separate run were pre-processed using Satori 1.8 (Brain Innovation, the Netherlands). Our pre-processing pipeline included the following steps: raw data conversion to optical density, data quality assessment by removing channels with Scalp Coupling Index (SCI) lower than 0.75 (Pollonini et al., 2014), temporal filtering (High-pass (Butterworth) = 0.01; Low pass = 0.50 Hz), motion correction with the Temporal Derivative Distribution Repair (TDDR) method and spike removal; physiological noise filtering using short-channel regression (Brigadoi and Cooper, 2015; Tachtsidis and Scholkmann, 2016; Yücel et al., 2021), conversion from optical density data to concentration changes in oxygenated (HbO) and deoxygenated (HbR) haemoglobin, and data normalization (z-transformation).

When a participant had a channel with less than one run from each social condition with good data quality, the data from that channel was excluded, and consequently excluded for the dyad. If a dyad had fewer than two channels with good data quality that dyad was excluded from subsequent analysis, ensuring each included dyad had data from all considered ROIs. Following these criteria, approximately 80% of the channels were included (TPJ = 81%, dlPFC = 74%, frontopolar = 86%).

#### 2.6.3 Interbrain synchrony analyses

Wavelet transform coherence (WTC) was used to derive IBS values for each dyad, using the Wavelet Transform Coherence Toolbox (Grinsted et al., 2004) for (MathWorks 2017). We calculated the WTC for each pair of corresponding channels for every run of each dyad and coherence data outside of the cone of influence were excluded. The coherence values were then averaged over the 12 s corresponding to the imagery period were then averaged (considering a 4 s delay for the hemodynamic response; Benerradi et al., 2023; Cinciute, 2019) across the 0.15–0.32 Hz frequency band (~3 to 6.5 period seconds, Nguyen et al., 2021). Coherence values were averaged per valence and social condition, resulting in 6 coherence values per channel per dyad (3 valences x 2 social conditions).

### 2.7 Statistical analyses

All statistical analyses were conducted on RStudio (version 2023.12.1+402), using the ARTool package (Wobbrock et al., 2011) for running ANOVAs on non-parametric data (after testing for normality) by applying the Aligned Rank Transform to the data beforehand; the *glmmTMB* package (Brooks et al., 2017) to run general linear mixed models (GLMM), as WTC data follows a beta distribution (0 to 1) and these models allow for data nesting (Nguyen et al., 2021); the *emmeans* package (Lenth, 2024) or *post hoc* analysis (pairwise comparisons) and its *emtrends* function to obtain correlations (and contrasts between correlations) between IBS and behavioral/psychological measures. Multiple comparisons in *post hoc* analyses were corrected using the False Discovery Rate (FDR) method (Benjamini and Hochberg, 1995).

#### 2.7.1 Individual behavioral measures

Variations in subjective valence ratings across valences and social conditions were analysed using a non-parametric ANOVA model after testing for normality with the Shapiro-Wilk test, to validate the presented situations' valence and to test for differences across social conditions and between mothers and children. The model included valence rating as the output, and valence type, social condition and subject (mother/child) as fixed and interacting factors (see model 1 in Supplementary material). Valence ratings were calculated as the average valence rating for each valence in each social condition for each participant (6 values per participant), Participant ID was not included as a random intercept to prevent model overfitting/singularity issues.

#### 2.7.2 Psychological measures

We calculated the means and standard deviations for each subscale from the ERQ-CA and the DERS-SF. Associations between all variables were calculated using Spearman correlations, and reliability measures were obtained with Cronbach's alpha.

#### 2.7.3 IBS

To address our research question of whether IBS varied as a function of valence and social condition, w e tested for IBS variation across different valences, social conditions, and ROIs using a GLMM model (see model 2 in the Supplementary material). IBS was added as the output, and valence, social condition, and ROI as fixed and interacting factors. Dyad ID was added as a random intercept, and valence, social condition, and ROI were added as random slopes. To control for potential confounding effects, we fitted an additional GLMM including children's biological sex and age (z-scored), as well as mothers' age (z-scored), as main effects (see model 2 in the Supplementary material).

#### 2.7.4 IBS and psychological/behavioural measures

Additionally, we investigated whether IBS during situations imagined *with each other* varied as a function of both we tested if mothers individual (psychological measures) and dyadic measures (behavioral measures). First,' difficulties in ER during negative affect were associated with IBS during negative situations by fitting a GLMM model that included the z-scores from the DERS-SF and ROI as a fixed and interacting effects (see model 3 in the Supplementary material). Second, to assess whether IBS varied with children's use of different ER strategies, we ran a GLMM model adding the z-scores for both ERQ subscales as fixed factors. Each subscale was also added as an interacting factor with valence type and ROI (see model 4 in Supplementary material). Finally, we tested whether IBS varied as a function of dyadic subjective valence ratings. For the behavioral models, we tested two GLMM models, one including the z-scores from dyadic average and the other including the difference valence rating as fixed and interacting factors (see models 5 and 6 in the Supplementary material). GLMM models were only performed with the data from the conditions were significant IBS variation was observed. Moreover, the GLMM model testing the association between mothers' DERS-SF and IBS in particular, only included the negative condition, as this questionnaire is focused on difficulties in emotion regulation for negative affect. Similarly to the 2.7.3 IBS analyses, we ran additional GLMM models to control for children's biological sex and age, and for mothers' age (see models 3.1, 4.1, 5.1 and 6.1 in the Supplementary material).

## 3 Results

### 3.1 Subjective valence ratings

We tested whether the situations presented for imagery were perceived with the intended valence, and if the perceived valence varied with social condition and/or between mothers and children. For this, variations in individual subjective valence ratings were tested with an ANOVA model (mixed effects), with type of valence, social condition and subject as fixed and interacting effects. The model revealed significant main effects of valence [(*F*_(2, 420)_ = 1,108.200, *p* = 2.220 × 10^−16^)] and social condition [(*F*_(1, 420)_ = 22.059, *p* =3.247 × 10^−6^)], a significant double interaction between social condition and valence [(*F*_(2, 420)_ = 9.618, *p* = 8.241 × 10^−5^)], and between subject and valence [(*F*_(2, 420)_ = 4.540, *p* = 0.011)], and a significant triple interaction between valence, social condition and subject [(*F*_(2, 420)_ = 5.333, *p* = 0.005)]. The remaining effects and interactions were non-significant. *Post hoc* analyses for investigating the three-way interaction was performed by examining the effect of valence within each social condition and subject level showed that, in both social conditions, mothers and children rated positive situations significantly higher than neutral (mothers: *p*_*with each other*_ < 0.001, *p*_*without each other*_ < 0.001; children: *p*_*with each other*_ < 0.001, *p*_*without each other*_ < 0.001) and negative ones (mothers: *p*_*with each other*_ < 0.001, *p*_*without each other*_ < 0.001; children: *p*_*with each other*_ < 0.001; *p*_*without each other*_ < 0.001), with neutral situations also rated significantly higher than negative ones (mothers: *p*_*with each other*_ < 0.001, *p*_*without each other*_ < 0.001; children: *p*_*with each other*_ < 0.001, *p*_*without each other*_ < 0.001). These results indicated that stimuli were perceived according to the intended valence (Table 1).

**Table 1:** Mother and child average subjective valence ratings (based on the SAM scale) for each valence type in each condition. ± standard deviation.

Subject	Without each other			With each other		
	Negative	Positive	Neutral	Negative	Positive	Neutral
Child	1.875 ± 0.839	7.375 ± 1.320	5.372 ± 0.639	2.418 ± 1.317	7.980 ± 0.741	5.453 ± 0.548
Mother	1.886 ± 0.791	7.505 ± 0.648	5.333 ± 0.399	1.762 ± 1.054	8.333 ± 0.692	5.474 ± 0.542

0.05.

**Figure 4:**
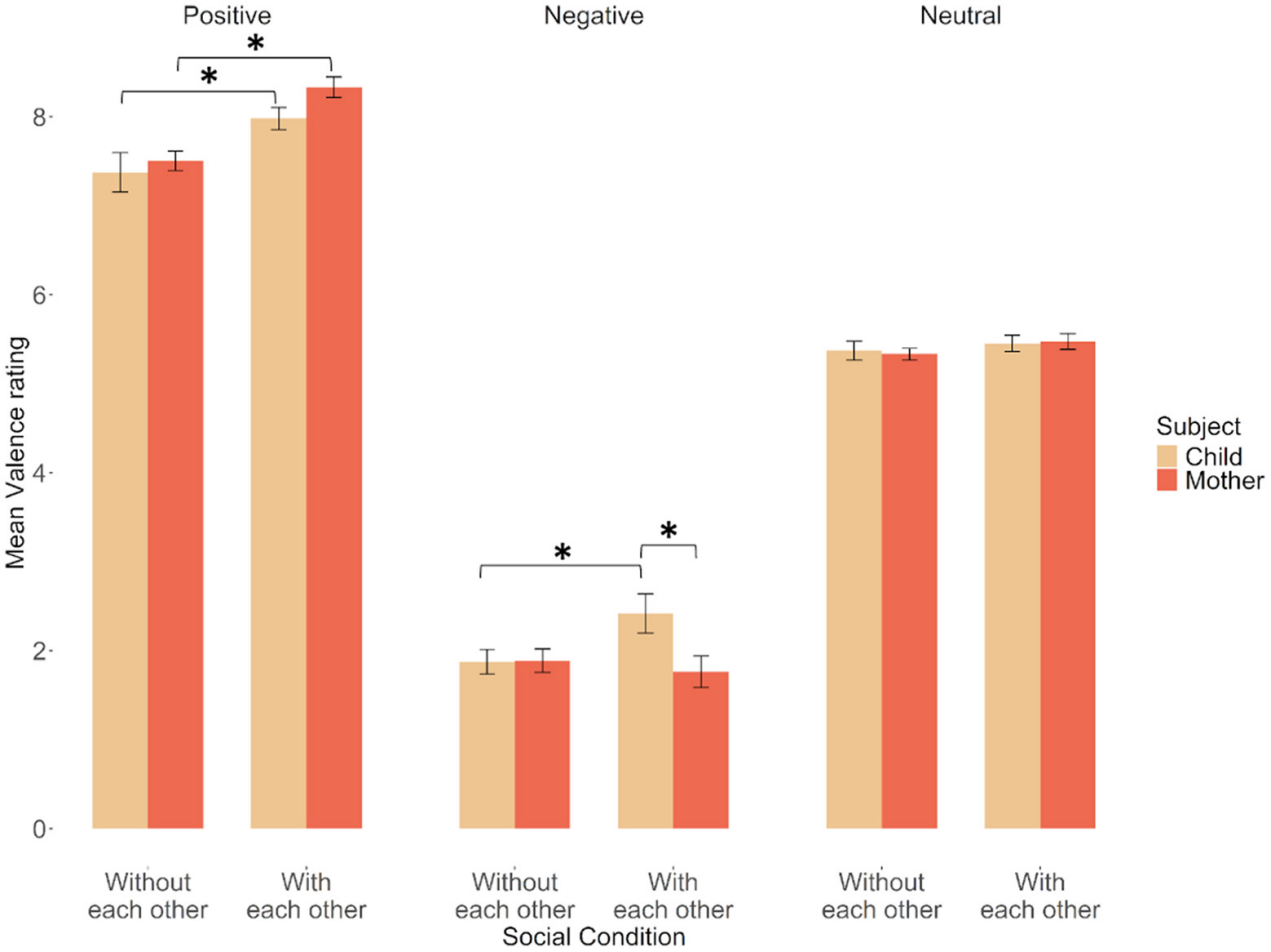
Mean SAM scores for all valences in both social conditions. Subjective valence ratings were analysed using an ANOVA model that included valence, social condition, and subject as factors. The analysis revealed a significant three-way interaction among valence, social condition, and subject. The figure displays the average valence ratings reported by mothers and children for each emotional valence under both social conditions. Statistically significant differences between mothers' and children's ratings, as well as between social conditions for the same valence, are indicated in the figure. Error bars indicate the standard error of the mean. *p < 0.05.

Furthermore, when considering the effect of social condition within each valence and subject level, we observed that both mothers and children rated positive situations in the *with each other* condition significantly higher than in the *without each other* condition (*p*_mothers_ < 0.001; *p*_children_ = 0.015), and that children rated negative situations imagined *with each other* higher than imagined *without each other* (*p* = 0.019). The latter pattern was not present for mothers (*p* = 0.580) and subjective valence ratings for neutral situations did not significantly differ between social conditions (*p*_mothers_ = 0.419; *p*_children_ = 0.580). Finally, comparing mothers' and children's subjective ratings, we observed that children rated negative situations imagined *with each other* significantly less negative than mothers (*p* = 0.004), with no other differences observed between mothers and children (Table 1; Figure 4). These results showed that positive situations imagined *with each other* were reported as more positive by both mothers and children. In contrast, only children rated negative situations as less negative in the *with each other condition*.

### 3.2 Psychological measures

Descriptive statistics for DERS-SF and ERQ subscales, as well as Cronbach's alpha values, can be found in the Supplementary Materials, Supplementary Table 2. No significant correlations were found between the questionnaire subscales (Supplementary Table 3).

### 3.3 IBS

**Figure 5:**
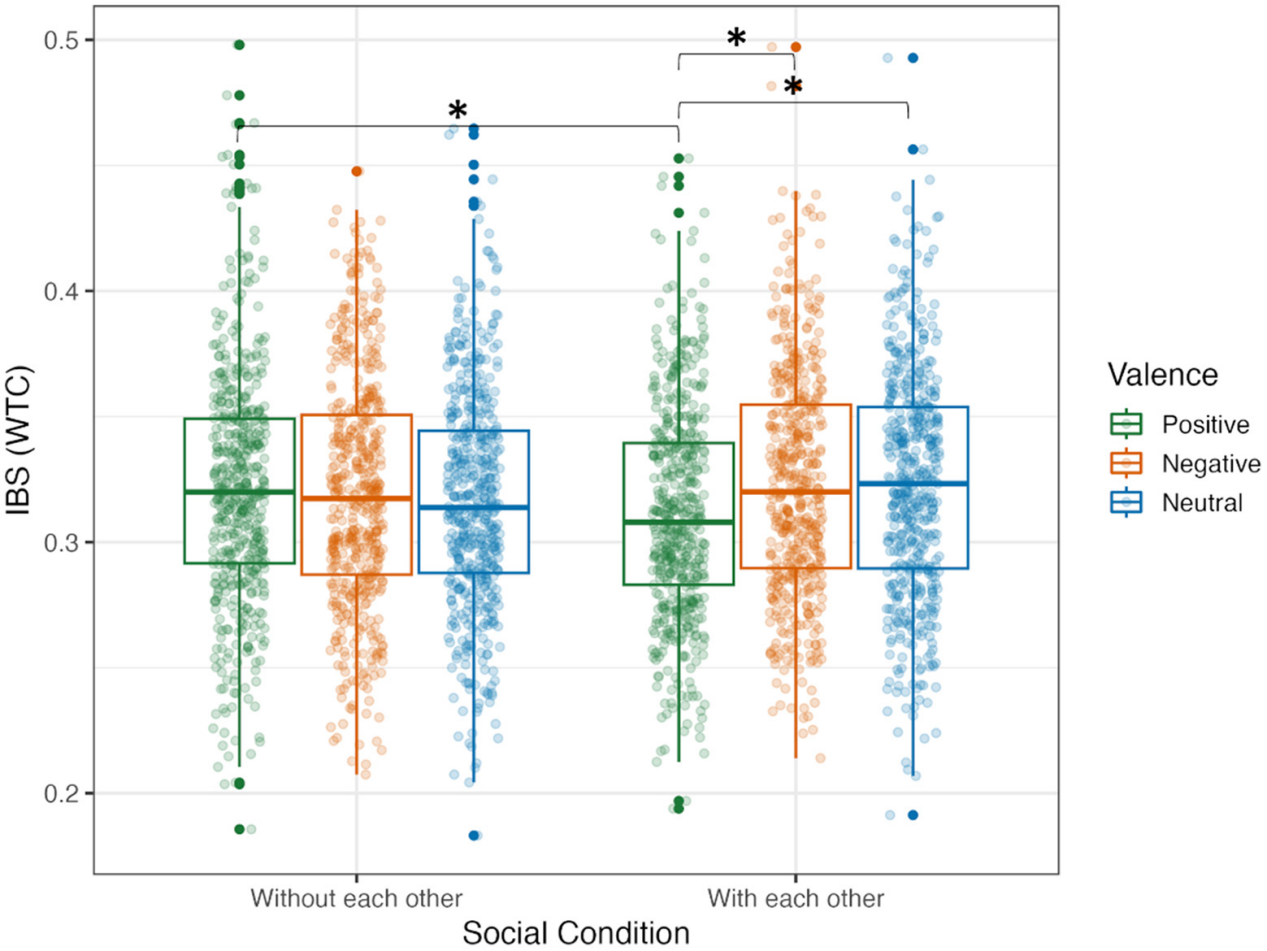
IBS in mother-child dyads according to social condition and valence during emotional imagery. Differences in IBS were tested by adding valence, social condition and ROI to the GLMM analyses, with results showing a significant interaction between valence and social condition. *Post hoc* pairwise comparisons showed a significant higher IBS for negative and neutral situations imagined with each other compared to positive ones, and a higher IBS for positive situations imagined without each other compared to imagined without each other. IBS values were derived using WTC. Box plot show the median and interquartile range for each valence in each social condition. Dots represent individual data points. *p ≤ 0.05.

We conducted a GLMM model to test if IBS was modulated as a function of valence type, social condition, and ROI. This model revealed a significant interaction between valence and social condition (*X*^2^(2) = 21.356, *p* = 2.305 x 10^−5^). All other main effects and interactions tested remained non-significant. *Post hoc* analyses for the interaction between valence and social condition, when isolating the factor valence, showed significant differences between valences only in the *with each other* social condition. In this condition, IBS was significantly higher in negative (*EMMs* = 0.324*, SE* = 0.003, *p* < 0.001) and neutral (*EMMs* = 0.323*, SE* = 0.003, *p* = 0.5) situations compared to positive ones (*EMMs* = 0.312*, SE* = 0.003) (Figure 5). Additionally, when isolating the factor social condition, *post hoc* results showed that IBS was significantly higher for positive situations in the *without each other* (*EMMs* = 0.322*, SE* = 0.003) than in the *with each other* (*EMMs* = 0.312*, SE* = 0.003, *p* = 0.009) condition. No other *post-hoc* contrasts reached significance. The interaction between valence and social condition in the GLMM remained significant after controlling for children's biological sex and for mothers' and children's age, χ^2^(2) = 21.353, p = 2.308 × 10^−5^. *Post hoc* analyses yielded the same results after controlling for these variables: *with each other* IBS higher in negative (*EMMs* = 0.324*, SE* = 0.003, *p* < 0.001) and neutral (*EMMs* = 0.322*, SE* = 0.003, *p* = 0.5) compared to positive situations (*EMMs* = 0.312*, SE* = 0.003), and IBS in positive situations imagined *without each other* (*EMMs* = 0.321*, SE* = 0.003) higher than without each other (*EMMs* = 0.312*, SE* = 0.003, *p* = 0.009). Additionally, we observed a significant main effect of mothers' age (X^2^(2) = 3.867, *p* = 0.049), with *post hoc* analyses showing a negative association (*trend* = −0.017, *SE* = 0.009, 95% *CI* = [−0.034–5.63 x 10^−5^]).

### 3.4 IBS link to behavioral and psychological measures

#### 3.4.1 Psychological measures—mother's DERS-SF

**Figure 6:**
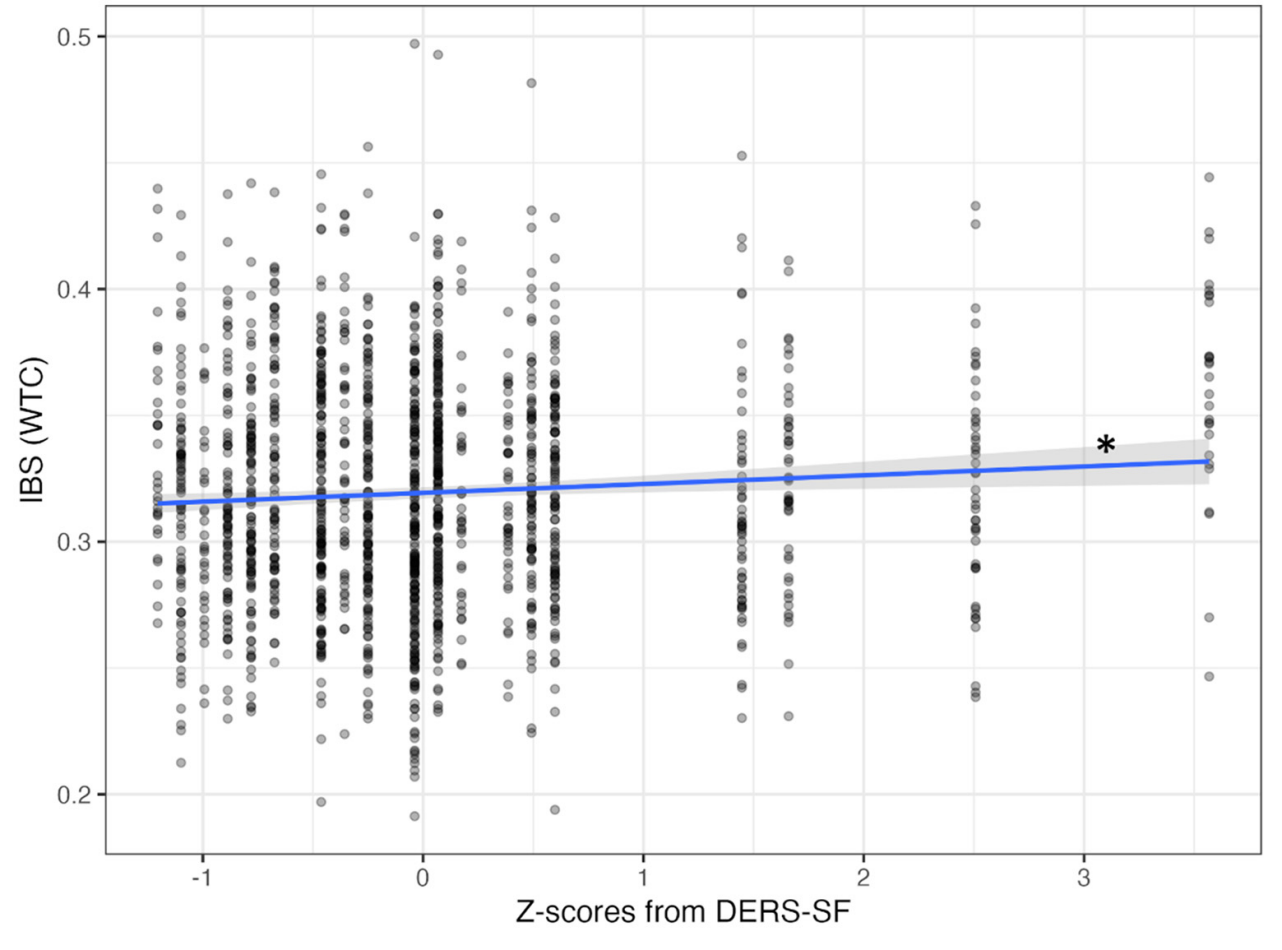
IBS variation according to mothers' DERS-SF scores (z-scores) for the negative valence across ROIs in the with each other social condition. The GLMM model revealed a significant main effect of the DERS-SF scores. Higher z-scores represent increased emotion regulation difficulties. Dots represent individual data points. *p < 0.05.

We tested the hypothesis that IBS during negative situations imagined *with each* other varied as a function of mothers' difficulties in emotion regulation during negative affect, as measured by DERS-SF. To examine this association, *z*-scored DERS-SF values were included as a factor in the GLMM model assessing IBS for negative valence in the *with each other* condition. The results showed a significant effect for DERS-SF scores (*X*^2^(1) = 5.325, *p* = 0.021) but no other significant main effects or interactions. *Post hoc* analysis showed a positive association between IBS and DERS-SF scores (trend = 0.027, SE = 0.013, 95% CI = [0.001 0.053]). These results show that IBS in the negative valence across ROIs tends to increase in dyads where mothers score higher on difficulties in emotion regulation (Figure 6). After controlling for the effects of children's biological sex, children's and mothers' age, the significant effect of DERS-SF scores remained significant (*X*^2^(1) = 6.122, *p* = 0.013), with a positive association between IBS and DERS-SF scores [*trend* = 0.029, *SE* = 0.013, 95% *CI* = (0.003 0.055)].

#### 3.4.2 Psychological measures—child's ERQ

**Figure 7:**
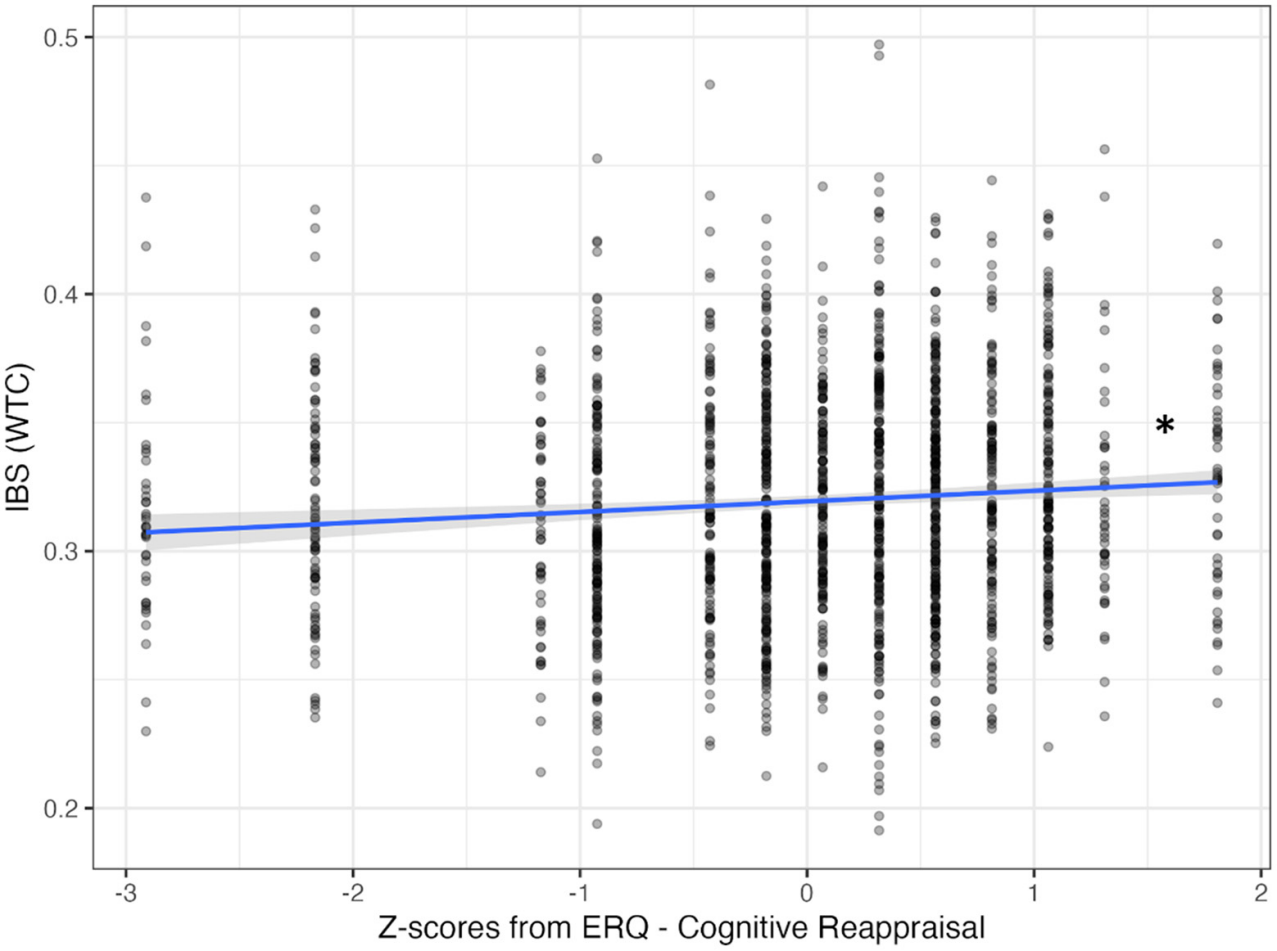
Association between IBS and children's use of cognitive reappraisal (ERQ—Cognitive reappraisal) according to valence in the with each other social condition. The GLMM model included valence, ROI and z-scores from the ERQ—Cognitive reappraisal and showed a significant main effect of ERQ—Cognitive reappraisal. The x-axis represents the z-scores from the ERQ—Cognitive reappraisal, where greater scores represent a greater use of this regulation strategy. Dots represent individual data points. * *p* < 0.05.

We tested the hypothesis that IBS during situations imagined *with each other* was associated with children's use of different ER strategies by running an additional GLMM model. Z-scored scores for the two ERQ subscales (Cognitive reappraisal and Expression suppression) were included as predictors to assess their relation with mother-child IBS. The results showed a significant main effect of valence (*X*^2^(2) = 20.803, *p* = 3.039 x 10^−5^) and of Cognitive Reappraisal (*X*^2^(1) = 5.641, *p* = 0.018), with no other fixed or interacting factors reaching statistical significance. *Post hoc* analyses revealed a positive correlation between IBS and Cognitive Reappraisal [*trend* = 0.020, *SE* = 0.009, 95% *CI* = (0.002 0.038)] across all valences and ROIs in the *with each other* social condition (Figure 7). These results showed that mother-child IBS across all valences and ROIs increased the more children used Cognitive Reappraisal as an ER strategy. After controlling for childrne's biological sex and age, as well as mothers' age, the effects of valence (*X*^2^(2) = 20.594, *p* = 3.374 x 10^−5^) and of Cognitive Reappraisal (*X*^2^(1) = 7.511, *p* = 0.006) remained significant, with a positive association between IBS and Cognitive Reappraisal [*trend* = 0.023, *SE* = 0.009, 95% *CI* = (0.005 0.051)]. The main effect of mothers' age was also significant (*X*^2^(1) = 3.962, *p* = 0.045), with *post hoc* showing a negative association [*trend* = −0.018, *SE* = 0.009, 95% *CI* = (−0.035 −2.7 x 10^−5^)].

#### 3.4.3 Behaviour

To investigate whether IBS varied with dyadic subjective emotional experiences in the *with each other* social condition we ran two additional GLMM models including valence and ROI as predictors. One model included z-scored values of dyadic average, while the second included z-scores from difference valence ratings between mother and child, to assess their respective association with IBS. Only the main effect of valence was significant (average ratings: *X*^2^(2) = 8.121, *p* = 0.017; differences in valence ratings: *X*^2^(2) = 18.876, *p* = 7.963 × 10^−5^). Mean and standard deviation for dyadic mean subjective valence ratings and differences in subjective valence ratings can be found in the Supplementary Material (Supplementary Table 4). When controlling for children's biological sex and age, and mothers' age, valence remained significant (average ratings: *X*^2^(2) = 8.467, *p* = 0.015; differences in valence ratings: *X*^2^(2) = 18.744, *p* = 8.506 × 10^−5^).

## 4 Discussion

This study investigated whether IBS between mothers and their children depending on valence (positive, negative and neutral) and social condition (*with each other* vs. *without each other*) during imagined emotional situations. Furthermore, we aimed to investigate if such mother-child IBS modulation was associated with individual differences in mothers' difficulties in ER and children's use of different ER strategies.

Imagining the presence of close others can be comforting under distress, particularly when the relationship quality is high, with social presence being previously shown to modulate neural activity (for a review, see Long et al., 2020). As imagery supports anticipation and preparation of possible future events (Holmes et al., 2008), interpersonal imagery might provide insight into how cognitive neural processes converge or diverge between individuals when imagining living emotional situations experienced alone or shared with other, and how such differences relate to personal expectations and perceived interaction quality.Previous studies have focused on the effects of emotional content on IBS (e.g., Azhari et al., 2020), and even on the association between IBS and individual differences in ER skills (Reindl et al., 2018). However, how IBS varies across valence and social contexts when both mothers and their children are equally engaged by the emotional content of a task, and its association with their ER skills/strategies, has not been assessed. The findings from our study showed that IBS was significantly higher during negative and neutral situations than positive ones only during the *with each other* condition. Moreover, when testing the association between IBS and mothers' individual differences in difficulties in ER for the negative valence in the *with each other condition*, we observed increased IBS in dyads with mothers who reported more difficulties in ER. Regarding children's use of different ER strategies, we observed that, during the *with each other* social condition, dyads with children who self-reported to make greater use of the cognitive reappraisal strategy (ERQ-CA) for ER, showed higher IBS during imagined emotional experiences, regardless of valence.

Mothers and children rated their emotional subjective experiences after each imagery period. Subjective valence ratings of imagined positive situations indicated that both mothers and children reported feeling more positively when imagining living those situations *with each other*, as compared to *without each other*. In other words, both participants reported feeling better imagining living positive situations together. Such findings are consistent with previous research showing that children report to feel better when their parents are present (Morris et al., 2017), and that sharing positive experiences with others increases emotional positivity more generally (Lambert et al., 2013; Reis et al., 2017). Our study extends these findings by showing that social sharing in the context of parent-child imagined positive experiences also yields increased positivity. In contrast, in negative contexts, children reported feeling less negatively when imagining living the situations together with their mothers. This suggests that the imagined presence of the mother in negative scenarios might have buffered children's subjective emotional responses. This finding is consistent with the literature identifying parents as primary sources of emotional support during pre-adolescence, with children relying on them in times of distress (Kerns and Brumariu, 2018; Koehn and Kerns, 2016). Our study extends these findings by showing that the *imagined* presence of the mother during negative experiences can also have a comforting effect. Moreover, it also reinforces the validity of imagery as an ecological approach to study emotional regulation neural mechanisms in children.

However, this effect was not observed in mothers. From the mothers' perspective, negative situations imagined *with* or *without* their children were rated similarly. This might indicate that mothers did not derive emotional comfort from their children's presence when both of them were affected by the negative situation. This interpretation aligns with the developmental trajectory of the parent–child relationship, which, during middle childhood, is still evolving toward reciprocity but remains mainly characterised by the parents' role as the emotional support provider (Kerns and Brumariu, 2018; Koehn and Kerns, 2016). Overall, these findings suggest that while both mothers and children experienced enhanced positive affect when imagining sharing positive experiences, only children experience emotional relief while sharing negative experiences.

IBS between mothers and children during imagined emotional situations was found to vary as a function of both the social condition and valence across all ROIs (right frontopolar, dlPFC, and TPJ) but only so during situations imagined *with each other*. For those situations, IBS was significantly lower during positive situations than during negative and neutral ones.

Overall, IBS variation associated with physical presence has been previously observed in studies where the same tasks were performed with participants in the same or separate rooms (Miura and Noguchi, 2022), including during exposure to emotional stimuli (Azhari et al., 2020). The present study extends these findings by showing that the imagined presence of another can also modulate IBS, specifically in emotional contexts, and particularly so for the mother-child dyad with children ages 10 to 14. Moreover, a higher IBS during negative (as compared to positive) contexts has been previously observed in sequential fMRI studies using paradigms involving the viewing of emotional films (Nummenmaa et al., 2012) and speaking/listening to emotionally expressive language (Smirnov et al., 2019). This study extends such findings by showing that increased IBS during negative (vs. positive) emotional experiences also occurs when participants imagine experiencing the same situations, where they were both the direct target of all situations, together, and by measuring IBS using fNIRS hyperscanning.

When considering dyadic mean valence ratings and dyadic differences in valence ratings, we observed that positive situations imagined *with each other* were not only rated as more positive by mother-child dyads, but also showed greater neural agreement in perceived positivity compared to the *without each other* social context. Although speculative, the Broaden-and-Build Theory of Positive Emotions (Fredrickson, 2001) might be considered as a possible framework for interpreting lower IBS during positive experiences. This theory suggests that negative emotions tend to narrow attentional focus, thought, and behavioral repertoires, whereas positive emotions broaden these processes, potentially leading to greater dyadic cognitive variability during imagined positive scenarios. Evidence also suggests that positive emotions broaden attention and cognition relative to neutral states, while support for negative emotions narrowing these processes compared to neutral states remains limited (Fredrickson and Branigan, 2005). Thus, imagining emotional situations *with each other* may generally elicit broader content than imagining them *without each other*, with variability likely higher in positive contexts and more constrained in negative or neutral ones.

Further insight may come from the Irruption Theory (Froese et al., 2024), which proposes that IBS may be reduced during interactive social contexts compared to simultaneously attending to the same stimuli. This decreased synchrony is proposed to reflect increased individual neural entropy, *i.e*., variations in cognitive processing, reflecting the subjectivity that comes from interpersonal interactions, whereas shared stimulus exposure tends to align neural activity, potentially leading to increased IBS (Froese et al., 2024). Considering both frameworks, the observed decrease in IBS from positive situations imagined *without each other* to those imagined *with each other* might reflect a dual source of variability: interaction-related neural dynamics and the broader thought repertoire associated with positive emotions. These interpretations should, however, be taken with caution, as no significant association between IBS and behavioral measures was observed. Further research is needed to better understand these differences and their significance.

Mothers' difficulties in ER were observed to be positively associated with IBS during negative scenarios imagined *with each other*. A higher score on the DERS-SF indicates greater ER difficulties, including problems related to acceptance, clarity, impulse control, engaging in goal-directed behaviors, and accessing ER strategies during negative experiences (Kaufman et al., 2016; Moreira et al., 2022). Similar patterns have been observed in other contexts. For instance, Azhary and colleagues (2022) using fNIRS hyperscanning, observed that parenting stress is positively associated with IBS in the left prefrontal regions during parent-child shared play, possibly reflecting an increased engagement of social-cognitive processes (including attention and planning) for these dyads to engage in shared play. A positive association between ER difficulties and synchrony was also observed during a dyadic movement task, suggesting that while increased synchrony might reflect enhanced positive affect, it can simultaneously reflect increased reliance on co-regulation rather than self-regulation (Galbusera et al., 2019). It might thus be that dyads in which mothers report more difficulties in ER during negative experiences may require a greater cognitive effort when imagining shared negative emotional scenarios, potentially reflected in greater similarity in neuronal cognitive processing in areas related to attention, ER and mentalizing. Maternal challenges in managing negative emotions might therefore influence the neural mechanisms underlying how mother and child perceive shared negative experiences, highlighting the importance of parental ER skills, as they serve as both models and support figures for children (Morris et al., 2017).

When investigating the association between IBS and children's use of ER strategies during emotional experiences imagined *with each other*, our results showed that IBS was positively associated with children's use of cognitive reappraisal, regardless of valence or ROI. In other words, mother-child dyads in which children indicated making greater use of cognitive reappraisal showed unspecifically increased IBS. As an adaptive ER strategy, cognitive reappraisal involves the reframing of emotional situations in a way that changes their emotional impact, which is usually associated with enhanced positive and diminished negative affect (Gullone and Taffe, 2012). Previous research, based on heart rate variability, has shown that, when children are better at regulating their own emotions, they are more likely to show sympathy and feel less distressed by others' emotions (Fabes et al., 1993). Similarly, studies on parent-child IBS during cooperative tasks have shown that IBS is higher when parents and children exhibit stronger ER skills (Reindl et al., 2018). Our findings build on this literature by showing that children's use of cognitive reappraisal is specifically associated with increased mother-child IBS during imagined shared emotional experiences. This suggests that, when both individuals are the target of emotional situations, children's greater use of more adaptive ER strategies may be reflected by enhanced mother-child neural attunement. This neural attunement might be a reflection of parental modelling and reinforcement of ER strategies (Morris et al., 2017). Consequently, greater IBS might reflect a dyadic preference for cognitive reappraisal, and thus similar cognitive processes. Further research focused on mothers' preferred ER strategy is needed to clarify whether the positive association between IBS and ER strategy use reflects parental influence on children's ER strategy selection. It is important to note that, unlike the DERS-SF (Kaufman et al., 2016; Moreira et al., 2022), the ERQ questionnaire (Carvalho, 2014; Gullone and Taffe, 2012) evaluates children's use of different ER strategies, thereby reflecting the strategies children employ rather than how effectively they employ them.

### 4.1 Limitations and future directions

This study examined mother–child IBS during imagined shared emotional experiences, exploring its association with both maternal and child ER skills and use across three emotional valences and two social conditions. While the emotional scenarios were designed to evoke distinct emotions within each valence category (e.g., sadness, fear, happiness and pride), participants rated their experiences solely in terms of in a scale that allowed to dissociate negative from positive and neutral affect following each imagery period. This approach allowed validation of the stimulus-based valence. However, no additional data were collected regarding the specific type of emotions experienced. Future research could include assessments of discrete emotions (e.g., anger, frustration, happiness, pride), which may provide more insights into how emotion is associated with IBS variability. Investigating these alongside dimension could help clarify whether IBS is modulated by specific affective states rather than valence alone.

Additionally, although the study manipulated imagined social presence, all dyads were physically co-present throughout the task. This raises the possibility that physical presence may have influenced IBS independently of the imagined social condition, although this effect remained constant across conditions. To explore these effects, future studies could replicate the paradigm with physical separation as an additional variable. This design would allow a better understanding of the role of physical co-presence in IBS variation during imagined emotional interactions.

The frequency band selected for IBS analyses partially overlapped with the respiration range. Although short-channel regression was applied to minimizing noise derived from physiological signals, including respiration, it cannot be entirely ruled out as a potential confounding factor. The relatively small sample size of this study might also impact our results. We used a within-subjects design to minimise this constraint by reducing between-participant variability. Nevertheless, future studies with larger samples may be valuable to assess the generalisation of the findings here reported.

Finally, our findings are based on imagined situations, either with or without the presence of the partner. While this paradigm allow us to investigate IBS in the absence of physical synchrony/cues, it may differ from real-life experiences, especially interpersonal interactions. Imagery relies on internally generated representations shaped by previous experiences and individual expectations (Cocquyt and Palombo, 2023), imagined interaction may thus vary more across individuals. In contrast, real interactions involve reciprocal behavior between the partners, which constrains responses and may influence IBS (Hasson et al., 2012). Future studies might benefit from investigating IBS during real shared emotional experiences to better understand how these differ from imagined ones, and how those differences might be related to individual expectations.

## 5 Conclusion

Our study shows that IBS during imagined emotional situations directly targeting mothers and their children is modulated by valence and social condition. More specifically, IBS during situations imagined with each other was higher during negative and neutral situations than positive ones. These results extend previous findings by showing that the imagined presence of close others can modulate dyadic IBS as a function of stimulus valence, paving the way to further investigation of the neural mechanism contributing to the development of key interpersonal interaction skills. In line with this, in this study, we also observed an association between IBS in emotional situations and mothers' ER difficulties and children's use of cognitive reappraisal, suggesting that how similarly imagined shared emotional situations are perceived is influenced by individual ER processes.

## Data Availability

The raw data supporting the conclusions of this article will be made available by the authors, without undue reservation.

## References

[B1] Azhari A. Bizzego A. Esposito G. (2021). Father-child dyads exhibit unique inter-subject synchronization during co-viewing of animation video stimuli. Soc. Neurosci. 16, 522–533. doi: 10.1080/17470919.2021.1970016 34407724

[B2] Azhari A. Bizzego A. Esposito G. (2022). Parent–child dyads with greater parenting stress exhibit less synchrony in posterior areas and more synchrony in frontal areas of the prefrontal cortex during shared play. Soc. Neurosci. 17, 520–531. doi: 10.1080/17470919.2022.2162118 36576051

[B3] Azhari A. Gabrieli G. Bizzego A. Bornstein M. H. Esposito G. (2023). Probing the association between maternal anxious attachment style and mother-child brain-to-brain coupling during passive co-viewing of visual stimuli. Attach. Hum. Dev. 25, 19–34. doi: 10.1080/14616734.2020.1840790 33357029

[B4] Azhari A. Leck W. Q. Gabrieli G. Bizzego A. Rigo P. Setoh P. . (2019). Parenting stress undermines mother-child brain-to-brain synchrony: a hyperscanning study. Sci. Rep. 9:47810. doi: 10.1038/s41598-019-47810-4 31388049 PMC6684640

[B5] Azhari A. Lim M. Bizzego A. Gabrieli G. Bornstein M. H. Esposito G. (2020). Physical presence of spouse enhances brain-to-brain synchrony in co-parenting couples. Sci. Rep. 10:63596. doi: 10.1038/s41598-020-63596-2 32371912 PMC7200679

[B6] Benerradi J. Clos J. Landowska A. Valstar M. F. Wilson M. L. (2023). Benchmarking framework for machine learning classification from fNIRS data. Front. Neuroergonomics 4:994969. doi: 10.3389/fnrgo.2023.994969 38234474 PMC10790918

[B7] Benjamini Y. Hochberg Y. (1995). Controlling the false discovery rate: a practical and powerful approach to multiple testing. J. R. Stat. Soc. Ser. B Methodol. 57, 289–300. doi: 10.1111/j.2517-6161.1995.tb02031.x

[B8] Bradley M. M. Lang P. J. (1994). Measuring emotion: the self-assessment manikin and the semantic differential. J. Behav. Ther. Exp. Psychiatry 25, 49–59. doi: 10.1016/0005-7916(94)90063-9 7962581

[B9] Brigadoi S. Cooper R. J. (2015). How short is short? optimum source–detector distance for short-separation channels in functional near-infrared spectroscopy. Neurophotonics 2:025005. doi: 10.1117/1.NPh.2.2.025005 26158009 PMC4478880

[B10] Brooks M. E. Kristensen K. van Benthem K. J. Magnusson A. Berg C. W. Nielsen A. . (2017). glmmTMB balances speed and flexibility among packages for zero-inflated generalized linear mixed modeling. R J 9, 378–400. doi: 10.32614/RJ-2017-066

[B11] Carvalho M. D. (2014). Universidade De Lisboa Faculdade De Psicologia Auto-Regulação Emocional Em Crianças E Adolescentes Com E Sem Diabetes. Lisbon: Universidade de Lisboa.

[B12] Cinciute S. (2019). Translating the hemodynamic response: why focused interdisciplinary integration should matter for the future of functional neuroimaging. PeerJ. 7:6621. doi: 10.7717/peerj.6621 30941269 PMC6438158

[B13] Cocquyt C. M. Palombo D. J. (2023). Emotion in the mind's eye: imagination for adaptive cognition. Ann. N. Y. Acad. Sci. 1526, 59–72. doi: 10.1111/nyas.15011 37344351

[B14] Cole P. M. Martin S. E. Dennis T. A. (2004). Emotion regulation as a scientific construct: methodological challenges and directions for child development research. Child Dev. 75, 317–333. doi: 10.1111/j.1467-8624.2004.00673.x 15056186

[B15] Costa V. D. Lang P. J. Sabatinelli D. Versace F. Bradley M. M. (2010). Emotional imagery: assessing pleasure and arousal in the brain's reward circuitry. Hum. Brain Mapp. 31, 1446–1457. doi: 10.1002/hbm.20948 20127869 PMC3620013

[B16] De Felice S. Hakim U. Gunasekara N. Pinti P. Tachtsidis I. Hamilton A. (2024). Having a chat and then watching a movie: how social interaction synchronises our brains during co-watching. Oxf. Open Neurosci. 3:kvae006. doi: 10.1093/oons/kvae006 38707237 PMC11069416

[B17] Fabes R. A. Eisenberg N. Eisenbud L. (1993). Behavioral and physiological correlates of children's reactions to others in distress. Dev. Psychol. 29, 655–663. doi: 10.1037//0012-1649.29.4.655

[B18] Feldman R. (2015). The adaptive human parental brain: implications for children's social development. Trends Neurosci. 38, 387–399. doi: 10.1016/j.tins.2015.04.004 25956962

[B19] Feldman R. (2017). The Neurobiology of Human Attachments. Trends Cogn. Sci. 21, 80–99. doi: 10.1016/j.tics.2016.11.007 28041836

[B20] Fredrickson B. L. (2001). The role of positive emotions in positive psychology: the broaden-and-build theory of positive emotions. Am. Psychol. 56, 218–226. doi: 10.1037//0003-066X.56.3.218 11315248 PMC3122271

[B21] Fredrickson B. L. Branigan C. (2005). Positive emotions broaden the scope of attention and thought-action repertoires. Cogn. Emot. 19, 313–332. doi: 10.1080/02699930441000238 21852891 PMC3156609

[B22] Froese T. Loh C. L. Putri F. (2024). Inter-brain desynchronization in social interaction: a consequence of subjective involvement? Front. Hum. Neurosci. 18:1359841. doi: 10.3389/fnhum.2024.1359841 38532790 PMC10963429

[B23] Galbusera L. Finn M. T. M. Tschacher W. Kyselo M. (2019). Interpersonal synchrony feels good but impedes self-regulation of affect. Sci. Rep. 9:14691. doi: 10.1038/s41598-019-50960-0 31604966 PMC6789117

[B24] Grinsted A. Moore J. C. Jevrejeva S. (2004). Application of the cross wavelet transform and wavelet coherence to geophysical time series. Nonlinear Process. Geophys. 11, 561–566. doi: 10.5194/npg-11-561-2004

[B25] Gross J. J. (ed.). (2014). Handbook of Emotion Regulation, 2nd Edn. New York, NY: The Guilford Press.

[B26] Gullone E. Taffe J. (2012). The emotion regulation questionnaire for children and adolescents (ERQ-CA): a psychometric evaluation. Psychol. Assess. 24, 409–417. doi: 10.1037/a0025777 22023559

[B27] Gvirts H.Z. Perlmutter R. (2020). What guides us to neurally and behaviorally align with anyone specific? a neurobiological model based on fnirs hyperscanning studies. Neuroscientist 26, 108–116. doi: 10.1177/1073858419861912 31296135

[B28] Hasson U. Ghazanfar A. A. Galantucci B. Garrod S. Keysers C. (2012). Brain-to-brain coupling: a mechanism for creating and sharing a social world. Trends Cogn. Sci. 16, 114–121. doi: 10.1016/j.tics.2011.12.007 22221820 PMC3269540

[B29] Hebart M. N. Dickter A. H. Kidder A. Kwok W. Y. Corriveau A. Wicklin C. V. . (2019). Things: a database of 1,854 object concepts and more than 26,000 naturalistic object images. PLoS ONE 14:545954. doi: 10.1101/545954 PMC6793944 31613926

[B30] Holmes E. A. Mathews A. Mackintosh B. Dalgleish T. (2008). The causal effect of mental imagery on emotion assessed using picture-word cues. Emotion 8, 395–409. doi: 10.1037/1528-3542.8.3.395 18540755

[B31] Ji J. L. Heyes S. B. MacLeod C. Holmes E. A. (2016). Emotional mental imagery as simulation of reality: fear and beyond—a tribute to peter lang. Behav. Ther. 47, 702–719. doi: 10.1016/j.beth.2015.11.004 27816082 PMC5112008

[B32] Kaufman E. A. Xia M. Fosco G. Yaptangco M. Skidmore C.R. Crowell S.E. (2016). The difficulties in emotion regulation scale short form (DERS-SF): validation and replication in adolescent and adult samples. J. Psychopathol. Behav. Assess. 38, 443–455. doi: 10.1007/s10862-015-9529-3 41522882 PMC12788809

[B33] Kerns K. A. Brumariu L. e. (2018). “Attachment in middle childhood,” in Handbook of Attachment: Theory, Research, and Clinical Applications, (eds.) J. Cassidy, and P.R. Shaver (New York, NY: P.R. The Guilford Press), 349–365.

[B34] Koehn A. J. Kerns K. A. (2016). The supervision partnership as a phase of attachment. J. Early Adolesc. 36, 961–988. doi: 10.1177/0272431615590231

[B35] Kosslyn S. M. Ganis G. Thompson W. L. (2001). Neural foundations of imagery. Nat. Rev. Neurosci. 2, 635–642. doi: 10.1038/35090055 11533731

[B36] Kurdi B. Lozano S. Banaji M. R. (2017). Introducing the open affective standardized image set (OASIS). Behav. Res. Methods 49, 457–470. doi: 10.3758/s13428-016-0715-3 26907748

[B37] Lambert N. M. Gwinn A. M. Baumeister R. F. Strachman A. Washburn I. J. Gable S. L. . (2013). A boost of positive affect: the perks of sharing positive experiences. J. Soc. Pers. Relatsh. 30, 24–43. doi: 10.1177/0265407512449400

[B38] Lang P. J. (1980). “Behavioral treatment and bio-behavioral assessment: computer applications,” in Technology in Mental Health Care Delivery Systems, eds. J. B. Sidowski, J. H. Johnson, and T A. Williams (Norwood, NJ: Ablex Publishing), 119–137.

[B39] Lenth R. V. (2024). Emmeans: Estimated Marginal Means, aka Least-Squares Means. Vienna: R Foundation for Statistical Computing.

[B40] Lindquist K. A. Satpute A. B. Wager T. D. Weber J. Feldman Barrett L. (2016). The brain basis of positive and negative affect: evidence from a meta-analysis of the human neuroimaging literature. Cereb. Cortex 26, 1910–1922. doi: 10.1093/cercor/bhv001 25631056 PMC4830281

[B41] Long M. Verbeke W. Ein-Dor T. Vrtička P. (2020). A functional neuro-anatomical model of human attachment (NAMA): insights from first- and second-person social neuroscience. Cortex 126, 281–321. doi: 10.1016/j.cortex.2020.01.010 32092496

[B42] Long Y. H. Chen C. S. Wu K. R. Zhou S. Y. Zhou F. X. Zheng L. F. . (2022). Interpersonal conflict increases interpersonal neural synchronization in romantic couples. Cereb. Cortex 32, 3254–3268. doi: 10.1093/cercor/bhab413 34849643

[B43] MathWorks (2017). MATLAB (Version R2017b) [Computer software]. Natick, MA: The MathWorksInc.

[B44] Miller J. G. Vrtička P. Cui X. Shrestha S. Hosseini S. M. H. Baker J. M. . (2019). Inter-brain synchrony in mother-child dyads during cooperation: an fnirs hyperscanning study. Neuropsychologia 124, 117–124. doi: 10.1016/j.neuropsychologia.2018.12.021 30594570 PMC6937429

[B45] Miura N. Noguchi S. (2022). The presence of adjacent others facilitates interpersonal neural synchronization in the left prefrontal cortex during a simple addition task. Sci. Rep. 12:16936. doi: 10.1038/s41598-022-16936-3 35879339 PMC9314338

[B46] Moreira H. Gouveia M. J. Canavarro M. C. (2022). A bifactor analysis of the difficulties in emotion regulation scale—short form (DERS-SF) in a sample of adolescents and adults. Curr. Psychol. 41, 757–782. doi: 10.1007/s12144-019-00602-5

[B47] Morgan J. K. Santosa H. Conner K. K. Fridley R. M. Forbes E. E. Iyengar S. . (2023). Mother–child neural synchronization is time linked to mother–child positive affective state matching. Soc. Cogn. Affect. Neurosci. 18:nsad001. doi: 10.1093/scan/nsad001 36715078 PMC9976748

[B48] Morris A. S. Criss M. M. Silk J. S. Houltberg B. J. (2017). The impact of parenting on emotion regulation during childhood and adolescence. Child Dev. Perspect. 11, 233–238. doi: 10.1093/scan/nsad001 36715078 PMC9976748

[B49] Nguyen T. Hoehl S. Vrtička P. (2021). A guide to parent-child fNIRS hyperscanning data processing and analysis. Sensors . 21:dr84h. doi: 10.31234/osf.io/dr84h 34199222 PMC8231828

[B50] Nguyen T. Kungl M. T. Hoehl S. White L. O. Vrtička P. (2024). Visualizing the invisible tie: linking parent–child neural synchrony to parents' and children's attachment representations. Dev. Sci. 27*:*e13504. doi: 10.31234/osf.io/gafz3 38523055

[B51] Nummenmaa L. Glerean E. Viinikainen M. Jääskeläinen I. P. Hari R. Sams M. (2012). Emotions promote social interaction by synchronizing brain activity across individuals. Proc. Natl. Acad. Sci. 109, 9599–9604. doi: 10.1073/pnas.1206095109 22623534 PMC3386135

[B52] Pollonini L. Olds C. Abaya H. Bortfeld H. Beauchamp M. S. Oghalai J. S. (2014). Auditory cortex activation to natural speech and simulated cochlear implant speech measured with functional near-infrared spectroscopy. Hear. Res. 309:84. doi: 10.1016/j.heares.2013.11.007 24342740 PMC3939048

[B53] Rainville P. Bechara A. Naqvi N. Damasio A. R. (2006). Basic emotions are associated with distinct patterns of cardiorespiratory activity. Int. J. Psychophysiol. 61, 5–18. doi: 10.1016/j.ijpsycho.2005.10.024 16439033

[B54] Raposo A. Vicens L. Clithero J. A. Dobbins I. G. Huettel S. A. (2011). Contributions of frontopolar cortex to judgments about self, others and relations. Soc. Cogn. Affect. Neurosci. 6, 260–269. doi: 10.1093/scan/nsq033 20478834 PMC3110426

[B55] Ratliff E. L. Kerr K. L. Cosgrove K. T. Simmons W. K. Morris A. S. (2022). The role of neurobiological bases of dyadic emotion regulation in the development of psychopathology: cross-brain associations between parents and children. Clin. Child Fam. Psychol. Rev. 25, 5–18. doi: 10.1007/s10567-022-00380-w 35113318 PMC9169725

[B56] Reindl V. Gerloff C. Scharke W. Konrad K. (2018). Brain-to-brain synchrony in parent-child dyads and the relationship with emotion regulation revealed by fNIRS-based hyperscanning. NeuroImage 178, 493–502. doi: 10.1016/j.neuroimage.2018.05.060 29807152

[B57] Reis H. T. O'Keefe S. D. Lane R. D. (2017). Fun is more fun when others are involved. J. Posit. Psychol. 12, 547–557. doi: 10.1080/17439760.2016.1221123 28919919 PMC5597001

[B58] Rodrigues I. Pereira J. Costa D. Correia R. Simões M. Direito B. . (2026). Modulation of interbrain synchrony by emotional valence and maternal presence in mother-child dyads: neural links to empathy and attachment. Sci. Rep. 16:13692. doi: 10.1038/s41598-026-43086-7 41840031 PMC13125508

[B59] Silk J. S. Steinberg L. Morris A. S. (2003). Adolescents' emotion regulation in daily life: links to depressive symptoms and problem behavior. Child Dev. 74, 1869–1880. doi: 10.1046/j.1467-8624.2003.00643.x 14669901

[B60] Smirnov D. Saarimäki H. Glerean E. Hari R. Sams M. Nummenmaa L. (2019). Emotions amplify speaker–listener neural alignment. Hum. Brain Mapp. 40, 4777–4788. doi: 10.1002/hbm.24736 31400052 PMC6865790

[B61] Stietz J. Jauk E. Krach S. Kanske P. (2019). Dissociating empathy from perspective-taking: evidence from intra- and inter-individual differences research. Front. Psychiatry 10. doi: 10.3389/fpsyt.2019.00126 30930803 PMC6428036

[B62] Tachtsidis I. Scholkmann F. (2016). False positives and false negatives in functional near-infrared spectroscopy: issues, challenges, and the way forward. Neurophotonics 3:031405. doi: 10.1117/1.NPh.3.3.031405 27054143 PMC4791590

[B63] Thompson K. I. Schneider C. J. Rocha-Hidalgo J. Jeyaram S. Mata-Centeno B. Furtado E. . (2024). Constructing the “family personality”: can family functioning be linked to parent–child interpersonal neural synchronization? *J. Pers*. 93, 755–766. doi: 10.1111/jopy.12973 39248009 PMC11890187

[B64] Travassos C. Sayal A. Direito B. Castelhano J. Castelo-Branco M. (2020). Volitional modulation of the left dlpfc neural activity based on a pain empathy paradigm—a potential novel therapeutic target for pain. Front. Neurol. 11:714. doi: 10.3389/fneur.2020.00714 32793103 PMC7394699

[B65] Wang S. Lu J. Yu M. Wang X. Shangguan C. (2022). “I'm listening, did it make any difference to your negative emotions?” evidence from hyperscanning. Neurosci. Lett. 788:136865. doi: 10.1016/j.neulet.2022.136865 36067901

[B66] Wobbrock J. O. Findlater L. Gergle D. Higgins J. J. (2011). “The aligned rank transform for non-parametric factorial analyses using only anova procedures,” in proceedings of the SIGCHI conference on human factors in computing systems, CHI'11. (*New York, NY:* Association for Computing Machinery*)*, 143–146. doi: 10.1145/1978942.1978963

[B67] Yücel M. A. Lühmann A. V. Scholkmann F. Gervain J. Dan I. Ayaz H. . (2021). Best practices for fNIRS publications. Neurophotonics 8:012101. doi: 10.1117/1.NPh.8.1.019802 33442557 PMC7793571

[B68] Zimeo Morais G. A. Balardin J. B. Sato J. R. (2018). FNIRS Optodes' Location Decider (fOLD): a toolbox for probe arrangement guided by brain regions-of-interest. Sci. Rep. 8, 1–11. doi: 10.1038/s41598-018-21716-z 29463928 PMC5820343

