# Supplementary material for: Emotion regulation in mother-child dyads is associated with interbrain synchrony during imagined shared emotional experiences
Source: Front Psychol. 2026 Jul 1;17:1813005. doi: 10.3389/fpsyg.2026.1813005 (PMC13368476; doi:10.3389/fpsyg.2026.1813005)
Supplement: Supplementary file 1 [file Table_1.DOCX]

Supplementary Material

# Supplementary Methods

## Electrodermal activity

### Set-up and data collection parameters

Physiological arousal was measured in terms of EDA. For that effect, before fNIRS cap placement, each participant was fitted with two electrodes between the digital and medial phalanges of the index and middle fingers from the non-dominant hand, using electrode gel (Gel101 from Biopac). The data was recorded simultaneously during the imagery task using a Biopac MP150 setup with two GSR100C modules (one for the mother and one for the child) with the Acqknowledge 5.0 software (Biopac Systems, Inc., USA), at sample frequency of 1KHz frequency, with low (10Hz) and high (DC) pass filtering.

### EDA Analysis and data quality assessment

EDA data was exported from Acqknowledge in the .*mat* format, and pre-processed in MATLAB (2024B) using the MATLAB-based software Ledalab (V3.4.9). Following conversion to the Ledalab format, motion artefacts were removed by pre-processing the data with a moving average filter. Subsequently, the data were down-sampled to 1Hz and analysed using the Continuous Decomposition Analysis (CDA) – which decomposes the EDA signal into phasic and tonic components (Benedek & Kaernbach, 2010). From this analysis, we derived phasic information from EDA, with the event-related response window set to 1 - 12s after stimulus onset (corresponding to the imagery period) and the minimum threshold for EDA peak detection set to 0.01 muS.

Data quality was assessed by visual inspection of the plotted skin conductance data, where runs with too much noise were excluded, as were periods of data with movement spikes in low-noise recordings. When a participant had data from fewer than four imagery periods per valence and per social condition, the corresponding data were excluded from the final EDA dataset. After data quality assessment, we included data from 34 mothers and 31 children, from the 36 included dyads (based on fNIRS data quality assessment).

### Arousal as measured by EDA

Skin conductance response (SCR) was used to obtain arousal levels by using the average phasic driver within the response window variable (CDA.SCR in Ledalab output) for all imagery periods. To normalise the data, we subtracted the SCR value from the 8s preceding the imagery period (fixation cross) from the SCR values of each imagery period. The fixation cross average phasic drivers were obtained by altering the response window to match the fixation cross duration. As was done for IBS and behavioural measures, we derived 6 arousal values (3 valences x 2 social conditions) for each participant, by averaging the values of all imagery periods for each valence in each social condition.

## Statistical Analyses

Specified below are the statistical models for behavioural and IBS measures, as well as for the association between IBS and behaviour and IBS and psychological measures, and EDA.

**Behaviour:**

1. ART model: Valence rating ~ Valence * Social Condition * Subject

**IBS:**

1. Full GLMM model: IBS ~ Social Condition * Valence * ROI +

(1+ Social Condition + Valence + ROI | Dyad)

2.1) Full GLMM model controlling for age and sex: IBS ~ Social Condition * Valence * ROI + Children’s Biological sex + Children’s Age + Mothers’ Age +

(1+ Social Condition + Valence + ROI | Dyad)

**IBS * behaviour/psychological measures:**

1. DERS GLMM: IBS ~ ROI * DERS total

+ (1 ROI || Dyad)

3.1) DERS GLMM controlling for age and sex:: IBS ~ ROI * DERS total + Children’s Biological sex + Children’s Age + Mothers’ Age

+ (1 ROI || Dyad)

1. ERQ GLMM: IBS ~ Valence + ERQ – Cognitive Reappraisal + ERQ – Expression Suppression + ROI + ROI:Valence + ROI:ERQ Cognitive Reappraisal + ROI:ERQ Expression Suppression + Valence:ERQ - Cognitive Reappraisal + Valence:ERQ - Expression Suppression + ROI:Valence:ERQ - Cognitive Reappraisal + ROI:Valence:ERQ - Expression Suppression

+ (1 + Valence + ROI|| Dyad)

4.1) ERQ GLMM controlling for age and sex:: IBS ~ Valence + ERQ – Cognitive Reappraisal + ERQ – Expression Suppression + ROI + ROI:Valence + ROI:ERQ Cognitive Reappraisal + ROI:ERQ Expression Suppression + Valence:ERQ - Cognitive Reappraisal + Valence:ERQ - Expression Suppression + ROI:Valence:ERQ - Cognitive Reappraisal + ROI:Valence:ERQ - Expression Suppression + Children’s Biological sex + Children’s Age + Mothers’ Age

+ (1 + Valence + ROI|| Dyad)

1. Dyadic average valence ratings GLMM: IBS ~ Valence * ROI * Dyadic average scores + (1 + Valence + Dyadic average scores|| Dyad)

5.1) Dyadic average valence ratings GLMM controlling for age and sex:: IBS ~ Valence * ROI * Dyadic average scores + Children’s Biological sex + Children’s Age + Mothers’ Age

+ (1 + Valence + Dyadic average scores|| Dyad)

1. Differences in valence ratings GLMM: IBS ~ Valence * ROI * Differences in scores +
2. + Valence + Differences in scores|| Dyad)

6.1) Differences in valence ratings GLMM controlling for age and sex:: IBS ~ Valence * ROI * Differences in scores + Children’s Biological sex + Children’s Age + Mothers’ Age

+ (1 + Valence + Differences in scores|| Dyad)

**EDA:**

1. ART model: *Mean score ~ Valence * Social Condition + (1|Participant)*

# Supplementary Results

Changes in arousal across valences and social conditions were tested with a non-parametric ANOVA (mixed effects) model, with valence and social condition as fixed and interacting factors, and participant ID as a random intercept.

Variations in physiological arousal (EDA) were tested with an ANOVA model, with valence and social condition as fixed and interacting effects. The results from the model, revealed no significant main effects of valence (*F*(2, 305) = 1.304, *p* = 0.272) and social condition (*F*(1, 305) = 0.437, *p* = 0.509), or of the interaction between valence and social condition (*F*(2, 305) = 1.782, *p* = 0.170). Means and standard deviations for each valence in each condition can be found in Supplementary Table 5.

# Supplementary Figures and Tables

## Supplementary Tables

Table 1 Channels’ main specificities according to the fOLD toolbox and ROI distribution.

| Channel | Area | Specificity (%) | Area | Specificity (%) | ROI |  |
| --- | --- | --- | --- | --- | --- | --- |
| 1 | Frontopolar Area (BA 10) | 54 | Orbitofrontal Area (BA 11) | 45 | Frontopolar |  |
|  |  |  |  |  |  |  |
| 2 | Frontopolar Area (BA 10) | 87 | Dorsolateral Prefrontal Cortex (BA 9) | 5 | Frontopolar |  |
|  |  |  |  |  |  |  |
| 3 | Frontopolar Area (BA 10) | 69 | Orbitofrontal Area (BA 11) | 22 | Frontopolar |  |
|  |  |  |  |  |  |  |
| 4 | Frontopolar Area (BA 10) | 72 | Dorsolateral Prefrontal Cortex (BA 9) | 17 | Frontopolar |  |
|  |  |  |  |  |  |  |
| 5 | Dorsolateral Prefrontal Cortex (BA 9) | 52 | Dorsolateral Prefrontal Cortex (BA 46) | 26 | dlPFC |  |
|  |  |  |  |  |  |  |
| 6 | Dorsolateral Prefrontal Cortex (BA 46) | 49 | Pars Triangularis Broca's (BA 45) | 32 | dlPFC |  |
|  |  |  |  |  |  |  |
| 7 | Frontopolar Area (BA 10) | 31 | Orbitofrontal Area (BA 11) | 30 | Frontopolar |  |
|  |  |  |  |  |  |  |
| 8 | Pars Triangularis Broca's (BA 45) | 44 | Dorsolateral Prefrontal Cortex (BA 46) | 43 | dlPFC |  |
|  |  |  |  |  |  |  |
| 9 | Dorsolateral Prefrontal Cortex (BA 9) | 62 | Frontopolar Area (BA 10) | 20 | dlPFC |  |
|  |  |  |  |  |  |  |
| 10 | Dorsolateral Prefrontal Cortex (BA 9) | 69 | Includes Frontal Eye Fields (BA 8) | 29 | dlPFC |  |
|  |  |  |  |  |  |  |
| 11 | Dorsolateral Prefrontal Cortex (BA 9) | 69 | Dorsolateral Prefrontal Cortex (BA 46) | 29 | dlPFC |  |
|  |  |  |  |  |  |  |
| 12 | Dorsolateral Prefrontal Cortex (BA 9) | 62 | Dorsolateral Prefrontal Cortex (BA 46) | 26 | dlPFC |  |
|  |  |  |  |  |  |  |
| 13 | Includes Frontal Eye Fields (BA 8) | 58 | Dorsolateral Prefrontal Cortex (BA 9) | 33 | dlPFC |  |
|  |  |  |  |  |  |  |
| 14 | Superior Temporal Gyrus (BA 22) | 35 | Middle Temporal Gyrus (BA 21) | 35 | TPJ |  |
|  |  |  |  |  |  |  |
| 15 | Superior Temporal Gyrus (BA 22) | 36 | Retrosubicular area (BA 48) | 19 | TPJ |  |
|  |  |  |  |  |  |  |
| 16 | Angular Gyrus (BA 39) | 35 | Superior Temporal Gyrus (BA 22) | 26 | TPJ |  |
|  |  |  |  |  |  |  |
| 17 | Angular Gyrus (BA 39) | 53 | Supramarginal Gyrus (BA 40) | 30 | TPJ |  |
|  |  |  |  |  |  |  |
| 18 | Angular Gyrus (BA 39) | 80 | V3 (BA 19) | 17 | TPJ |  |

Table 2 Means, standard deviations and Cronbach alpha values from the subscales of the DERS-SF and ERQ questionnaires.

| Subscale | Mean | S.D. | Min. - Max. | Cronbach alpha |
| --- | --- | --- | --- | --- |
| DERS-SF - Total | 1.824 | 0.628 | 1.067-4.067 | 0.927 |
| ERQ – Cognitive Reappraisal | 20.722 | 4.026 | 9.000-28.000 | 0.761 |
| ERQ – Expression Suppression | 10.889 | 2.734 | 4.000-17.000 | 0.742 |

Table 3 Spearman correlation values between the subscales of DERS-SF and ERQ. No statistically significant correlations were found.

|  | DERS-SF – Total | ERQ – Cognitive Reappraisal | ERQ – Expression Suppression |
| --- | --- | --- | --- |
| DERS-SF – Total |  | -0.062 | 0.124 |
| ERQ – Cognitive Reappraisal |  |  | 0.061 |

Table 4 Dyadic means valence ratings and dyadic differences in valence ratings for each valence of each social condition. ± standard deviation.

|  | Without each other | | | With each other | | |
| --- | --- | --- | --- | --- | --- | --- |
|  | Negative | Positive | Neutral | Negative | Positive | Neutral |
| Mean dyadic ratings | 1.881 ± 0.809 | 7.440 ± 1.034 | 5.353 ± 0.529 | 2.090 ± 1.229 | 8.157 ± 0.734 | 5.464 ± 0.541 |
| Differences in dyadic ratings | 0.771 ± 0.706 | 1.044 ± 1.021 | 0.428 ± 0.555 | 1.004 ± 0.949 | 0.582 ± 0.459 | 0.433 ± 0.446 |

Table 5 Means and standard deviations for arousal (in relation to the baseline) for both mother (n = 34) and child (n = 31), for the different valences and social conditions. Units in muS ± S.D).

| Without each other | | | With each other | | |
| --- | --- | --- | --- | --- | --- |
| Positive | Negative | Neutral | Positive | Negative | Neutral |
| -0.033 ± 0.183 | -0.001 ± 0.204 | -0.052 ± 0.150 | -0.018 ± 0.139 | -0.009 ± 0.223 | -0.013 ± 0.150 |

## Supplementary Figures

**
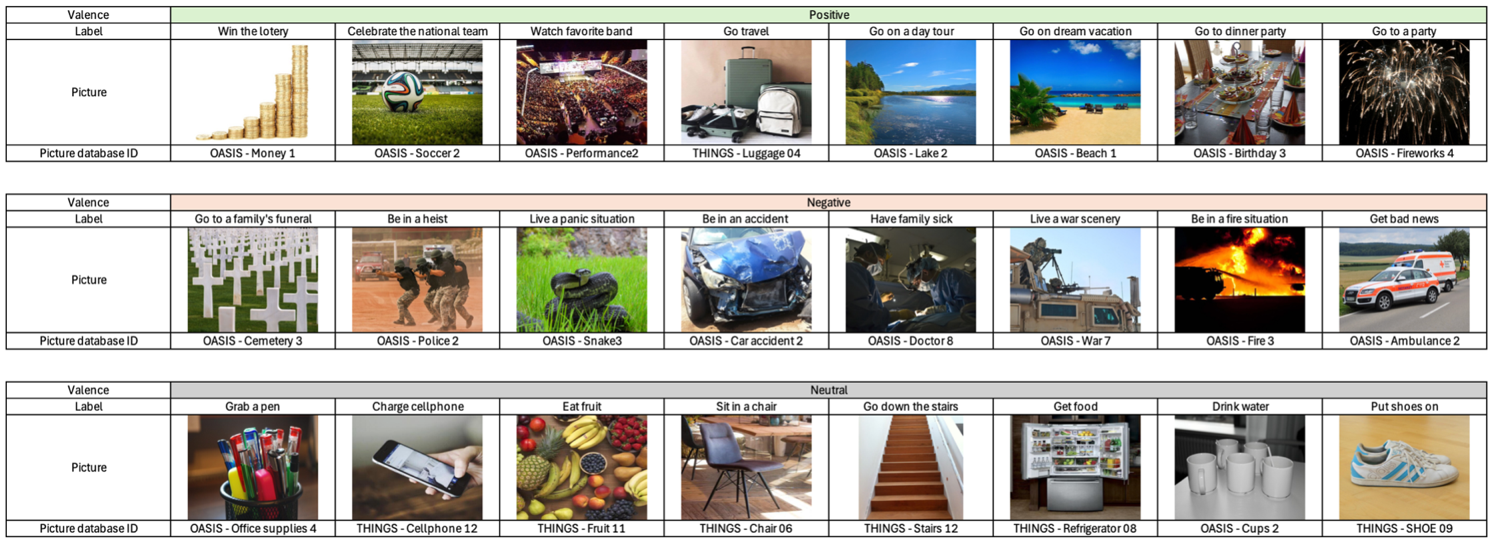
**

**Supplementary Figure 1.** Stimuli for the imagery task. Images were selected from the OASIS (Kurdi et al., 2017) and THINGS (Hebart et al., 2019) databases. Each stimulus label and database ID can be found above and below the corresponding picture, respectively. Stimuli are organised according to their valence.
